# Cortical actin nodes: Their dynamics and recruitment of podosomal proteins as revealed by super-resolution and single-molecule microscopy

**DOI:** 10.1371/journal.pone.0188778

**Published:** 2017-11-30

**Authors:** Yuki M. Shirai, Taka A. Tsunoyama, Nao Hiramoto-Yamaki, Koichiro M. Hirosawa, Akihiro C. E. Shibata, Kenichi Kondo, Atsushi Tsurumune, Fumiyoshi Ishidate, Akihiro Kusumi, Takahiro K. Fujiwara

**Affiliations:** 1 Institute for Frontier Life and Medical Sciences, Kyoto University, Kyoto, Japan; 2 Center for Meso-Bio Single-Molecule Imaging (CeMI), Institute for Integrated Cell-Material Sciences (WPI-iCeMS), Kyoto University, Kyoto, Japan; 3 Membrane Cooperativity Unit, Okinawa Institute of Science and Technology, Okinawa, Japan; 4 Olympus Corporation, Tokyo, Japan; 5 Nikon Instech, Tokyo, Japan; Oregon Health and Science University, UNITED STATES

## Abstract

Electron tomography of the plasma membrane (PM) identified several layers of cortical actin meshwork running parallel to the PM cytoplasmic surface throughout the PM. Here, cortical actin structures and dynamics were examined in living cells, using super-resolution microscopy, with (x,y)- and z-resolutions of ~140 and ~400 nm, respectively, and single-molecule imaging. The super-resolution microscopy identified sub-micron-sized actin clusters that appeared identical by both phalloidin post-fixation staining and Lifeact-mGFP expression followed by fixation, and therefore, these actin clusters were named “actin-pl-clusters”. In live cells, the actin-pl-clusters visualized by Lifeact-mGFP linked two or more actin filaments in the fine actin meshwork, acting as a node of the meshwork, and dynamically moved on/along the meshwork in a myosin II-dependent manner. Their formation depended on the Arp2/3 activities, suggesting that the movements could involve both the myosin motor activity and actin polymerization-depolymerization. The actin-pl-clusters differ from the actin nodes/asters found previously after latrunculin treatments, since myosin II and filamin A were not colocalized with the actin-pl-clusters, and the actin-pl-clusters were much smaller than the previously reported nodes/asters. The Lifeact linked to a fluorescently-labeled transmembrane peptide from syntaxin4 (Lifeact-TM) expressed in the PM exhibited temporary immobilization in the PM regions on which actin-pl-clusters and stress fibers were projected, showing that ≥66% of actin-pl-clusters and 89% of stress fibers were located in close proximity (within 3.5 nm) to the PM cytoplasmic surface. Podosome-associated cytoplasmic proteins, Tks4, Tks5, cortactin, and N-WASP, were transiently recruited to actin-pl-clusters, and thus, we propose that actin-pl-clusters also represent “actin podosome-like clusters”.

## Introduction

In recent years, the organization, dynamics, and functions of actin filaments on and near the cytoplasmic surface of the plasma membrane (PM), often termed cortical actin filaments or the cortical actin meshwork [1–5], have gained extensive attention. In the cortical actin meshwork, the meshwork apposed to the PM cytoplasmic surface, located within ~8 nm from the cytoplasmic surface, has been identified using three-dimensional (3D) reconstruction of electron microscopy (EM) images of PM specimens prepared by the rapid-freeze deep-etch technique [6,7]. This actin meshwork was termed the “actin-based membrane skeleton” [6,8,9], and similar meshwork structures have been observed in both the top (apical) and bottom (basal) PMs [9,10]. The actin-based membrane skeleton compartmentalizes the PM, inducing temporary confinement of almost all of the PM-associated/impregnated molecules for a few milliseconds to ~100 ms within a meshwork of ~40‒300 nm in diameter and hop diffusion owing to the movement of molecules from one compartment to an adjacent compartment [10–13]. Such temporary confinement could be important for enhancing signaling in a particular compartment [8, 14–16], which might be further enhanced by the oligomerization-induced trapping of receptors and their associated molecules [17].

The 3D-reconstituted EM images of the PM additionally clarified that actin filaments, mostly running parallel to the PM, exist within ~80 nm from the PM cytoplasmic surface, indicating the existence of several layers of actin meshwork along the PM. Xu et al. [2] found two layers of actin meshwork, each with an apparent thickness of 30‒40 nm, which were separated from each other by 50‒100 nm, using dual-objective STORM. Since the cells were extensively chemically fixed, extracted, and stained with Alexa647-phalloidin, the observed structures could have been induced by the extensive chemical crosslinking of several 8-nm-thick actin layers. Although the rapid freezing of samples using pure copper blocks cooled with liquid helium is likely to preserve the actin meshwork structure reasonably well [6,9], this discrepancy suggests that observing the cortical actin organization in living cells would provide more accurate information.

**Fig 1:**
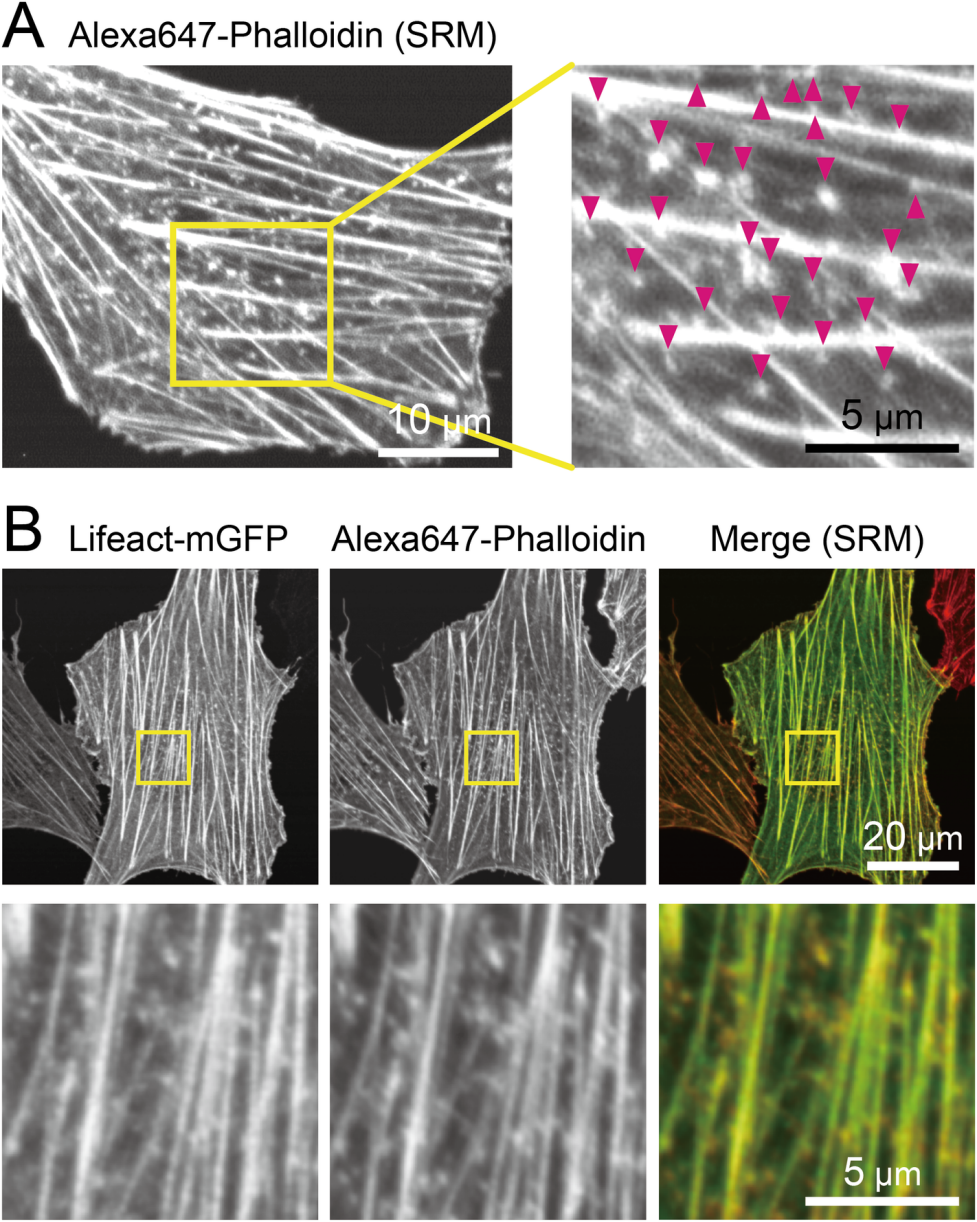
Actin-pl-clusters visualized by “p”halloidin and “l”ifeact in fixed NRK cells. **(A)** A representative confocal-based SRM (Olympus FV-OSR) image of cellular actin structures, including stress fibers, thinner filamentous mesh-like structures, and small actin clusters, visualized by Alexa647-phalloidin in NRK cells. The region within the yellow square is magnified on the right, with superimposed arrowheads showing elongated dot-like actin clusters. **(B)** A representative dual-color FV-OSR observation of NRK cells transfected with Lifeact-mGFP (green in the rightmost image) and stained with Alexa647-phalloidin (red). The corresponding region indicated by the yellow box in the top images is magnified in the bottom images.

One of the persisting key issues that must be addressed to understand the cortical actin-filament meshworks, particularly those located on the PM cytoplasmic surface, is whether the actin filaments in the meshwork are linked to each other by way of node-like structures. If such nodes exist, then their molecular composition, dynamics, and biological functions, and whether and how the nodes are related to dynamic actin asters, proposed previously [1,18,19], must be determined. After re-examining published 3D EM images [6,9,10], we were unable to identify such nodes. This may be a consequence of the vastly different spatial resolutions between fluorescence microscopy and electron microscopy, which generally differ by a factor of more than 100 (240 nm vs. 2 nm, respectively). If several actin filaments were assembled at a node, then, in fluorescence microscopy image, the node would appear as a large cluster, since fluorescence microscopy cannot resolve each individual actin filament near the node. However, electron microscopy allows each actin filament to be resolved very clearly, and thus a node (or a node-like structure) connecting actin filaments would simply appear to be a place where several actin filaments cross. Therefore, it was difficult to determine whether the apparent node found by fluorescence microscopy indeed represents a real structure or simply the incidental crossing of several actin filaments. However, these difficulties of identifying nodes in the 3D-reconstructed EM images suggest that the node structure linking the actin filaments must be small, with a diameter less than 20 nm, assuming that the cross-section diameter of actin filaments observed in the 3D EM images is approximately 8 nm. Furthermore, in several previous studies [1,18,19], actin nodes and asters were found or expected to be dynamic, owing to the motor action of myosin filaments. If the nodes connecting actin filaments move and if their movements could be visualized, then we would be able to identify the nodes and the actin filaments bound to the nodes. Such observations could be greatly enhanced by employing super-resolution microscopy, which has recently been used to observe actin filaments and other cellular structures near the PM in live and fixed cells [2,3,5,18,20,21]. However, in the studies where actin nodes, asters, stars, and/or vortices were found, the cells were generally pretreated with chemical inhibitors, including those that blocked actin polymerization [18,22] (however, in [18], the presence of actin nodes without latrunculin treatment was shown in panel E of Fig 1) or Arp2/3 function [21], or *in vitro* reconstituted actin-membrane systems were utilized, where myosin II and limited numbers of actin binding proteins were employed [19].

Therefore, the present study was undertaken with the following five objectives. (1) To detect actin nodes and node-like structures using super-resolution microscopy in living cells. (2) If actin nodes or node-like structures are found, to reveal 2a) their relationship with the cortical actin structures, 2b) their dynamics and possible causes of their dynamics, and 2c) their locations relative to the PM. (3) To clarify whether and how the detected actin clusters (nodes or node-like structures) are related to the actin nodes/asters found in latrunculin-treated cells and the *in vitro* actomyosin system formed on artificial membranes. (4) To discover molecules that colocalize with actin nodes and node-like structures, which might provide important information about their functions. (5) To clarify the relationship between the previously obtained 3D EM images of the cortical actin meshwork. Although the spatial resolutions of super-resolution microscopies are still >50× worse than those of 3D EM tomography, super-resolution microscopies have the critical advantage of enabling the observation of actin structures in living cells.

To achieve these five objectives, we employed commercially available super-resolution microscopes (SRMs) with spatial xy-resolutions of 115‒140 nm and a z resolution of ~400 nm and time resolutions of 0.5‒2.3 s. Until these SRMs became available, virtually none of the cortical actin-filament meshwork and actin-assembling nodes in living cells could be spatially resolved by conventional fluorescence microscopy, and thus our knowledge about these fine actin structures has been quite limited. No comprehensive study of these fine actin structures has been performed, even by total internal reflection fluorescence microscopy [TIRFM]. From the SRM images and videos published by Xu et al. [2], Luo et al. [18], Burnette et al. [20], Kiuchi et al. [3], and Li et al. [5], it was clear that some of the various actin structures and dynamics could be clarified even at the spatial resolutions of SRMs (*i*.*e*., worse by a factor of ~10‒100 as compared with electron microscopy). Therefore, we combined the SRMs with TIRFM and single-molecule observations (which employed TIRFM).

We paid special attention to the actin-concentrated submicron- and micron-level structures (with spatial resolutions of ~140 nm) and the actin filaments linked to these structures to detect actin “nodes” or “clusters.” Actin clusters and nodes have been proposed to be necessary for the formation and function of specialized membrane domains, formed by the cooperative interaction of the PM and actin filaments. For example, the cell-membrane fusion domains in fission yeast are induced by aster-like aggregates of short actin filaments, with their barbed ends focalized on the PM [4]. Dynamic nanoclusters of lipid-anchored raft-associated proteins can be induced to enhance their interactions by the active drive of actin asters, which are dynamically and continually formed by myosin II on the PM cytoplasmic surface [1]. Therefore, we examined whether actin binding and regulating proteins, filamin A, myosin II, cortactin, and N-WASP, as well as podosome-associated proteins, Tks4 and Tks5, are recruited to the actin nodes/clusters.

## Materials and methods

### Plasmid construction

The cDNAs encoding EGFP-cortactin, EGFP-MRLC, and EGFP-UtrCH were purchased from Addgene (#26722, #35680 and #26737, respectively). The cDNAs encoding Tks4 and Tks5 were purchased from Kazusa DNA Research Institute [23], amplified by PCR, and inserted into a plasmid encoding the Halo7-tag protein with a 45-base pair linker (15 amino acids with the sequence SGGGG ×3) between Halo7 and Tks4/Tks5. Halo7-N-WASP and Halo7-paxillin were generated by replacing the cDNA encoding the EGFP protein, in the EGFP-fused rat N-WASP plasmid (a kind gift from Dr. Jack Taunton, University of California San Francisco [24]) and the EGFP-paxillin plasmid [25], with the cDNA encoding the Halo7-tagged protein (Promega) and inserting a 45-base pair linker (15 amino acids, with the sequence SGGGG ×3). Halo7-filamin A was generated by replacing the cDNA encoding the mNeonGreen protein in the human mNeonGreen-filamin A plasmid (purchased from Allele Biotechnology) with the cDNA encoding the Halo7-tagged protein (Promega) and inserting a 45-base pair linker (15 amino acids, with the sequence SGGGG ×3).

Lifeact-mGFP was constructed by fusing the PCR-amplified sequence of Lifeact (MGVADLIKKFESISKEE) to the N-terminus of mGFP in the pEGFP-N1 vector [26]. The Lifeact-TM-ACP sequence (Lifeact-[SGGGG ×3]-Syntaxin4 [1-37/267-298]-[SGGGG ×3]-ACP) was generated as follows. The sequences encoding the 37 N-terminal amino acids (1‒37) and the 32 transmembrane amino acids (267‒298) of human syntaxin4 (a kind gift from Dr. Kazuhisa Nakayama, Kyoto University) were amplified by PCR. They were inserted into a pEGFP-N1 vector with the Lifeact sequence at the N-terminus and an extracellular ACP tag sequence at the C-terminus with an SGGGG ×3 linker.

### Cell culture, transfection, and fluorescence labeling

NRK cells were grown in Ham’s F12 medium (Sigma-Aldrich) supplemented with 10% fetal bovine serum (FBS; Sigma-Aldrich), 100 units/ml penicillin, and 0.1 mg/ml streptomycin (Gibco). They were transfected with various cDNAs by using a Nucleofector 2b Device (Lonza), according to the manufacturer’s recommendations. For microscopy experiments, cells were plated on glass-based dishes (Iwaki) and preincubated with 10 μg/ml fibronectin in Hanks’ balanced salt solution (HBSS; Nissui), buffered with 2 mM N-tris(hydroxymethyl)methyl-2-aminoethanesulfonic acid (TES; Dojindo), pH 7.4, for 20 min at 37°C. To fluorescently label Halo7-N-WASP, Halo7-Tks4, Halo7-filamin A, and Halo7-paxillin expressed in NRK cells (with co-expression of Lifeact-mGFP) with almost 100% (~50%) labeling efficiency, the cells were incubated with 30 nM (5 nM) tetramethylrhodamine (TMR)-conjugated Halo ligand (Promega) in Ham’s F12 medium containing 10% FBS, for 15 min at 37°C. After three washes with Ham’s F12 medium containing 10% FBS and buffered with 2 mM TES, pH 7.4 (HT medium), the cells were incubated for 10 min at 37°C in the same medium to remove the unreacted TMR-Halo ligand. Lifeact-TM-ACP expressed on the NRK cell surface was fluorescently labeled by incubating the cells with 0.8 μM phosphopantetheine transferase (Covalys) and 500 nM SeTau647-CoA (custom synthesized from SeTau647-maleimide [SETA BioMedicals] and CoA-SH [Covalys]) in HT medium for 5 min at 37°C, followed by three washes with HT medium. All microscopic observations were performed at 37°C. For the blebbistatin treatment, the cells were incubated with 25 μM blebbistatin (Sigma-Aldrich) in HT medium for 5 min at 37°C before observation. To observe the cells treated with latrunculin A (200 nM; Sigma-Aldrich) or CK-666 (50 and 200 μM; Sigma-Aldrich), microscopy observations were initiated immediately after the addition of the drugs in HT medium.

### Super-resolution microscopy observations

#### Olympus FV-OSR

NRK cells with or without Lifeact-mGFP expression were fixed with 4% paraformaldehyde in PBS at room temperature for 1 h, and washed three times with PBS. After an incubation with 0.1% Triton X-100 for 5 min and blocking with 5% skim milk for 1 h, the cells were stained with 500 nM Alexa647-phalloidin (Thermo Fisher Scientific) for 1 h, washed with PBS, and mounted in Permafluor medium (Thermo Fisher Scientific). Observations were performed with a 100×, 1.4 NA objective lens at room temperature using an Olympus FV-OSR system, relying on the reduced pinhole size and the software to enhance the high spatial frequency components. The pixel size of the final images is 43 nm.

#### Olympus SD-OSR

Observations of NRK cells expressing Lifeact-mGFP were performed with a 100×, 1.49 NA objective lens at 37°C using an Olympus SD-OSR system, relying on the spinning disk confocal based SRM (SDSRM, [27,28]), operated at 2 Hz (every 0.5 s, which is the same as the signal integration time) for 50 s. The pixel size of the final images is 40 nm.

#### Nikon N-SIM

Observations of NRK cells expressing Lifeact-mGFP were performed with a 100×, 1.49 NA objective lens at 37°C using a Nikon N-SIM microscope (3D-SIM mode; [29]), at a frame rate of 0.44 Hz (every 2.3 s, whereas the actual signal integration time was 0.1 s × 15 images = 1.5 s), for a period of 60 s. The pixel size of the final images is 32 nm.

#### Zeiss confocal microscope with Airyscan

NRK cells expressing Lifeact-mGFP after 4% paraformaldehyde fixation were observed with a 100×, 1.46 NA objective lens at room temperature, using the Airyscan-mode of a Zeiss LSM880 confocal microscope. The pixel size of the final images is 38 nm.

### Single fluorescent-molecule observations by TIRF microscopy

Fluorescently labeled molecules located on the ventral PM (which faces the coverslip) were observed at 37°C, using a custom-built objective lens-type TIRF microscope with simultaneous two-color image acquisition based on an inverted microscope (Nikon ECLIPSE Ti-E with 100×, 1.49 NA objective lens; 250× total magnification), as described previously [25,30,31]. The ventral PM was locally illuminated with an evanescent field (~50 μm in diameter). The fluorescence images of GFP/TMR [GFP/SeTau647] were separated by a 562-nm dichroic mirror (FF562Di03; Semrock) and were projected into two detection arms with bandpass filters of 500‒550 nm for GFP (FF01-525/50; Semrock) and 573‒613 nm for TMR (FF01-593/40; Semrock) [500‒550 nm for GFP (FF01-525/50; Semrock) and 665‒705 nm for SeTau647 (FF01-685/40; Semrock)]. The fluorescent images in each channel were projected onto a two-stage microchannel plate intensifier (C8600-03; Hamamatsu Photonics), coupled to a specially designed CMOS sensor-based camera (Photron) with an optical-fiber bundle, operated at 60 frames per second (fps). The pixel size of the final images is 68.0 nm. Although a simultaneous two-color microscope system was used, for ease of operation, alternation between multi-molecular observations of Lifeact-mGFP and single-molecule observations of cytoplasmic molecules labeled with TMR or transmembrane molecules labeled with SeTau647 was performed (mGFP first, then TMR or SeTau647). This procedure has an advantage in that we did not need to make any subtle adjustments of the laser power for each observation field, thus avoiding the leakage of the strong multi-molecular fluorescent signal of Lifeact-mGFP into the TMR or SeTau647 channel. All of the single-molecule spots in the obtained images were detected, and those that were detectable for durations longer than three frames (50 ms) were quantitatively analyzed [32,33].

### Western blotting

The cells were cultured in a 10-cm dish, and after the removal of the cell culture medium, 100 μl ice-cold PBS containing 0.5% protease inhibitor III (Calbiochem), 0.5% protease inhibitor VI (Calbiochem), and 1 mM EDTA was added, and then the cells were extracted with 25 μl of 5x sample buffer (312 mM Tris-HCl, pH 6.8, 10% SDS, 35% glycerol, 0.05% bromophenol blue, and 25% 2-mercaptoethanol). The whole extract was placed in boiling water for 1 min and centrifuged for 1 min, and the supernatant was subjected to polyacrylamide gel electrophoresis. The proteins were then electro-transferred to a polyvinyl difluoride membrane using a transfer apparatus, according to the manufacturer’s protocol (Bio-Rad). After an incubation with 3% skim milk in 150 mM NaCl buffered with 10 mM Tris-HCl (pH 7.4) for 30 min at room temperature, the membrane was incubated with 1 μg/ml antibody IgG against filamin A (rabbit, Cell Signaling Technology), Halo-tag protein (rabbit, Promega), α-tubulin (mouse, Abcam), or β-actin (mouse, Sigma-Aldrich) for 1 h, followed by an incubation with HRP-conjugated goat anti-mouse or anti-rabbit IgG (1:10,000; Jackson Immunoresearch Laboratories) for 1 h. The bands of the labeled proteins were then visualized using the Western BLoT substrate series (TAKARA BIO), according to the manufacturer’s protocol.

### Obtaining single-molecule trajectories and plots of MSD versus time

All of the actin-pl-clusters and membrane molecules observed in the images were used for analysis, without any arbitrary selection by the observers. The positions (x- and y-coordinates) of each cluster and membrane molecule were determined using a custom computer program, which uses the method developed by Gelles et al. [34]. For each trajectory, the mean-squared displacement (*MSD*) for every time interval was calculated according to the following formula [32,35]:

$$MSD(\Delta t)=MSD(n\delta t)=\frac{1}{N-1-n}\sum_{j=1}^{N-1-n}\left\{{\left[x(j\delta t+n\delta t)-x(j\delta t)\right]}^{2}+{\left[y(j\delta t+n\delta t)-y(j\delta t)\right]}^{2}\right\}$$

where *δt* is the frame time interval and (x(*jδt* +*nδt*), y(*jδt* +*nδt*)) describes the molecule’s position following a time interval Δ*t* = *nδt*, after starting at position (x(*jδt*), y(*jδt*)), *N* is the total number of frames in the recording sequence, and *n* and *j* are positive integers (*n* determines the time increment).

### Velocity estimation for the Actin-pl-clusters

The movements of actin-pl-clusters were analyzed based on the ensemble-averaged *MSD*-Δ*t* plot, which was obtained by averaging the *MSD* values over all actin-pl-clusters as a function of the frame time interval (Δ*t*). This was fitted by the following equations representing the mode of directed diffusion, in which a molecule moves in a direction at a constant drift velocity (*v*_x_, *v*_y_) with superimposed random movements according to the diffusion coefficient *D* [32,35]:

$$MSD(\Delta t)=4D\Delta t+{\left(v\Delta t\right)}^{2}$$

$$v^{2}={v_{x}}^{2}+{v_{y}}^{2}$$

### Defining the boundary of the actin-pl-clusters and stress fibers

Fluorescent images of Lifeact-mGFP (8-bit grayscale data, 300 × 300 pixels, 68.0 × 68.0 nm^2^/pixel) were binarized using adaptive (local) thresholding, and then threshold values were determined for each pixel [25,36,37]. The outermost row of white color pixels in a binary image was considered as the outline of an actin-pl-cluster or stress fiber zone, and was used to determine whether a single molecule was located inside or outside the actin-pl-cluster or stress fiber zone.

### TALL detection

Temporary Arrest of LateraL diffusion (TALL; coined by our research group in Shibata et al. [25], based on the term Stimulation-induced TALL (STALL) we originally proposed in Suzuki et al. [38, 39]) events were detected within each single-molecule trajectory by using the algorithm developed by Sahl et al. [40]. Parameters (detection circle radius and threshold residency time) were set based on the average diffusion coefficient of Lifeact-TM. The application of these parameters to the computer-generated simple-Brownian trajectories revealed false TALL events, representing 2‒5% of the total length of the trajectories. Using this program, all of the single-molecule trajectories obtained at 60 fps were classified into the three modes of motion: (1) the all-time mobile mode, (2) the mobile + TALL mode, and (3) the all-time immobile mode.

## Results

### Lifeact-mGFP and fluorescent phalloidin visualized similar actin clusters near the ventral PM in SR observations of fixed cells

In the present work, all of the super-resolution (SR) and single fluorescent-molecule observations were performed with the microscope focus placed at the PM facing the coverslip (ventral PM, facing the coverslip) using fibroblastic NRK cells, unless stated otherwise. First, the cellular actin structures were observed in fixed cells. For this purpose, the cells were chemically fixed with paraformaldehyde [41], stained with Alexa647-phalloidin, and then observed using a confocal-based SRM (Olympus FV-OSR, providing [x,y]- and z-resolutions of ~140 and 400 nm, respectively, at the fluorescence emission wavelength of 510 nm). A representative image is shown in Fig 1A. In addition to thick stress fibers and thinner filamentous (and sometimes mesh-like) structures, many small actin clusters with somewhat elongated dots were observed (indicated by arrowheads in Fig 1A). These elongated dots were observed using Alexa647-phalloidin staining, and thus they are likely to represent some form of actin clusters. Using conventional confocal fluorescence microscopy, a majority of these elongated actin clusters appeared as circular dots with a diffraction-limited size. Similar elongated dot-like structures were only occasionally found near the dorsal PM (PM facing the bulk culture medium, rather than the coverslip).

Next, we hoped to observe the behaviors of elongated actin clusters in living cells. However, since the actin filaments in live cells cannot be stained with fluorescent phalloidin, we considered using Lifeact-mGFP expressed in NRK cells. For this purpose, we next examined whether Lifeact-mGFP expressed in cells, after fixation with paraformaldehyde followed by staining with Alexa647-phalloidin, showed actin structures similar to those visualized with Alexa647-phalloidin (Fig 1B). Lifeact-mGFP also exhibited thick stress fibers, thinner filamentous structures, and many elongated-dot-like actin clusters. Importantly, virtually all of the actin clusters stained with Lifeact-mGFP were also stained with Alexa647-phalloidin, indicating that both fluorescent markers identified similar actin clusters. Importantly, the actin clusters detected by Alexa647-phalloidin staining in cells transfected with Lifeact-mGFP (Fig 1B, bottom-center) were also found in cells that had not been transfected with Lifeact-mGFP cDNA (Fig 1A). These results suggest that the exogenously expressed Lifeact-mGFP does not strongly and artifactually modify the organization of actin (as pointed out previously [42]), including the actin clusters. Thus, in the following experiments, Lifeact-mGFP was employed to monitor the behaviors of the actin clusters, and since they were marked by both “p”halloidin and “l”ifeact, we call the structures “actin-pl-clusters”.

### SRM observations of Lifeact-mGFP in live cells at time resolutions of 0.5 and 2.3 s

**Fig 2:**
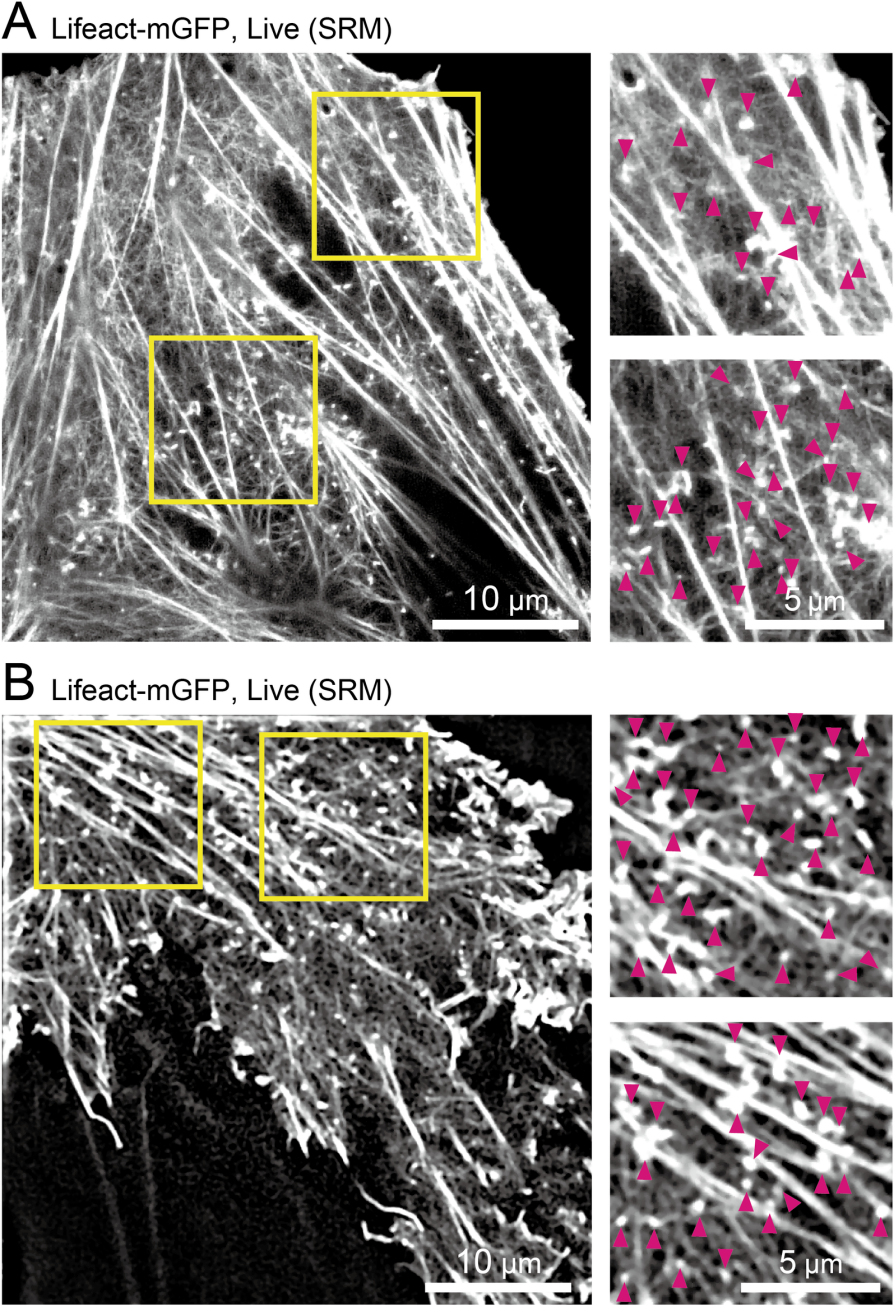
Live-cell super-resolution images of cortical actin structures, showing the existence of many actin-pl-clusters as well as the cortical fine actin meshwork and stress fibers. A representative snapshot from image sequences of NRK cells transfected with Lifeact-mGFP and observed by **(A)** a spinning-disk confocal-based SRM (SDSRM; Olympus SD-OSR system) operated at 2 Hz (a time resolution of 0.5 s with a signal integration time of 0.5 s) for a period of 50 s, and by **(B)** structured illumination microscopy (3D-SIM mode of Nikon N-SIM system) operated at 0.44 Hz (a time resolution of 2.3 s with a signal integration time of 0.1 s) for a period of 60 s. The regions within the yellow boxes in the images on the left are magnified on the right, with arrowheads showing actin-pl-clusters. For the original image sequences, see S1 Movie (SDSRM) and S2 Movie (3D-SIM).

We next observed actin-pl-clusters in living cells, using NRK cells expressing Lifeact-mGFP. To obtain ever-changing SRM images of Lifeact-mGFP in live cells, FV-OSR, which requires several minutes to obtain a single image, was considered to be too slow. Therefore, we used a spinning disk confocal based SRM (SDSRM; an Olympus SD-OSR system) operated at 2 Hz (every 0.5 s, which is the same as the signal integration time) for 50 s. A representative image is shown in Fig 2A. Stress fibers, fine actin filaments and their meshwork, and actin-pl-clusters (indicated by the magenta arrowheads in the magnified images on the right; the same images without arrowheads are shown in S1A Fig) could be observed, consistent with the results shown in Fig 1. Importantly, a thin actin filament meshwork could be clearly seen here, as compared with the images of the fixed cells shown in Fig 1, suggesting the importance of observing live cells. It is important to note that these observations were made in quiescent cells without any external stimulation.

Since SDSRM is a recently developed technique (although the principle has been known, it has seldom been implemented for actual biomedical applications), we next used a more established SRM method, structured illumination microscopy [29], to observe Lifeact-mGFP expressed in NRK cells. Importantly, the spatial resolutions of the two methods are considered to be approximately the same. The observations were made at a frame rate of 0.44 Hz (every 2.3 s, whereas the actual signal integration time was 0.1 s × 15 images = 1.5 s) for a period of 60 s, using a Nikon N-SIM microscope (3D-structured illumination microscopy [SIM] mode; Fig 2B). In Fig 2B, the actin organization in the ruffling membrane can be seen clearly in addition to stress fibers, fine actin filaments and their meshwork, and actin-pl-clusters (indicated by the magenta arrowheads in the magnified images on the right; the same images without arrowheads are shown in S1B Fig)

These results are consistent with previous observations using SRM, such as the observations in COS-7 cells using total internal reflection fluorescence (TIRF)-SIM and nonlinear SIM [5]. Therefore, the dynamics and molecular composition of uncharacterized actin-pl-clusters were next examined in detail.

**Fig 3:**
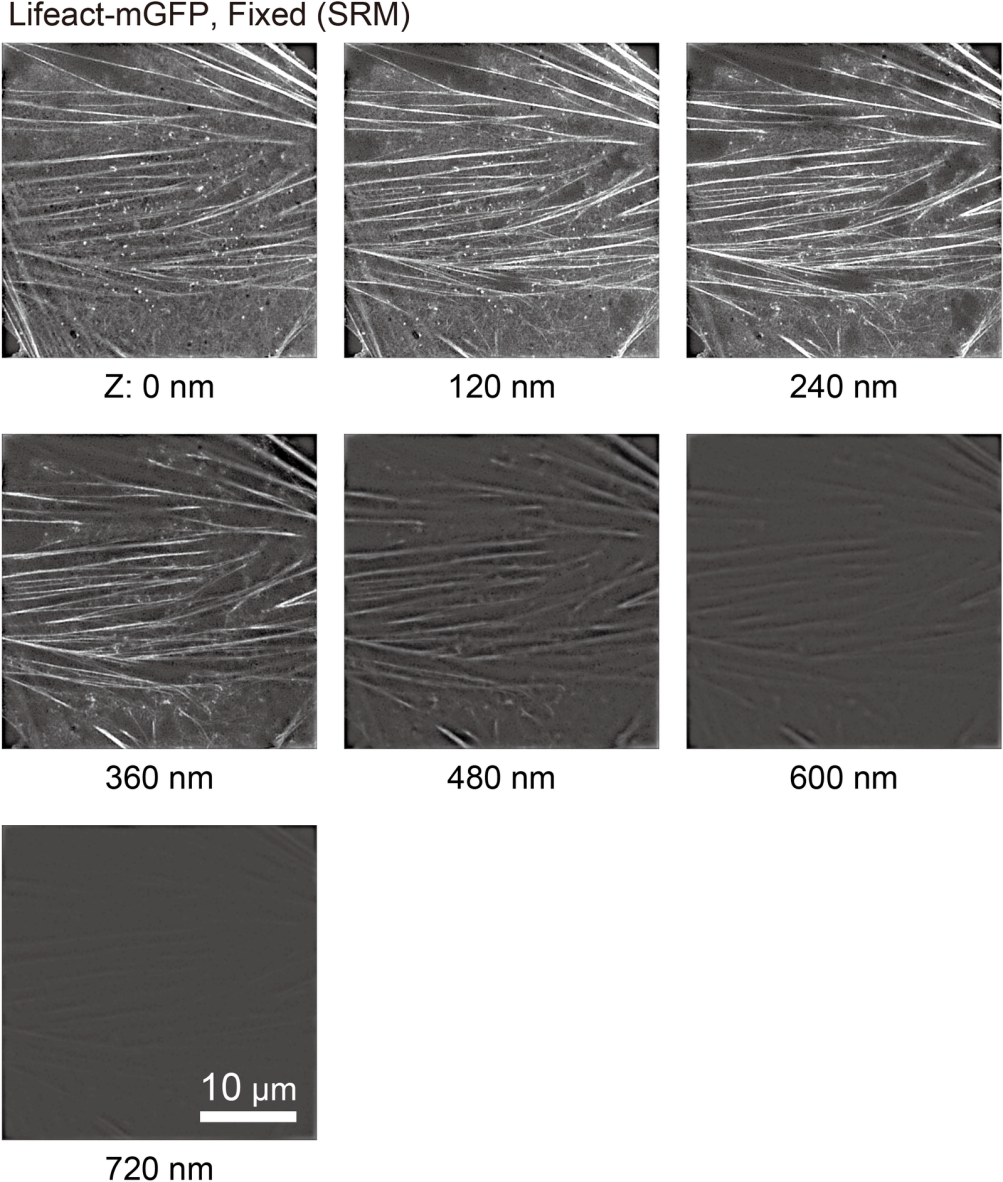
3D-SIM tomographic images of actin-pl-clusters in fixed NRK cells, showing that actin structures visualized by Lifeact-mGFP are localized within ~400 nm from the PM cytoplasmic surface. 3D-SIM observations of fixed NRK cells transfected with Lifeact-mGFP using a Nikon N-SIM system every 120 nm from the glass surface (Z: 0 nm) up to 720 nm, with z-resolution of ± 200 nm.

Images of Lifeact-mGFP obtained after fixation using 3D-SIM every 120 nm from the glass surface up to 720 nm (seven images with a z-resolution of ±200 nm) are shown in Fig 3. The overall signal intensity was maximal in the second image (‒80 nm~320 nm from the glass surface), which is consistent with the fact that the PM is located in this range, with a midpoint at 120 nm from the glass surface. Meanwhile, very little Lifeact-mGFP signal was observed in the images of 520~920 nm from the glass surface, showing that a large majority of actin filaments, including stress fibers, actin-pl-clusters, and the cortical actin meshwork, are located in the range of 0~400 nm from the PM (120~520 nm from the glass surface).

### Actin-pl-clusters often undergo morphological changes and translocations along the actin meshwork and lead to actin meshwork formation

**Fig 4:**
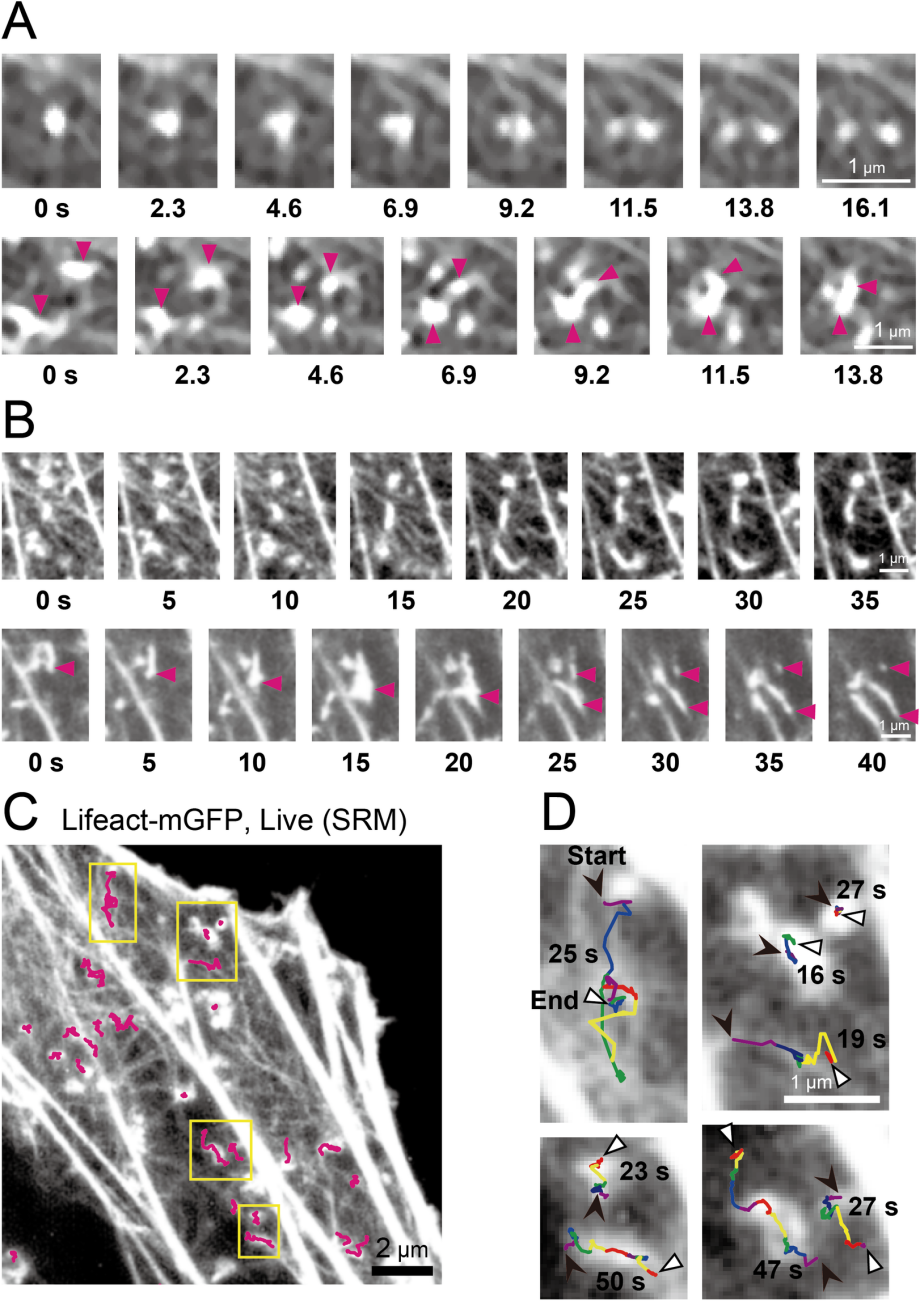
Actin-pl-clusters exhibited continuous dynamic morphological changes and movements on and along the cortical actin meshwork, linking actin filaments (acting as nodes in the meshwork). **(A)** Images of actin-pl-clusters visualized by Lifeact-mGFP were obtained using a Nikon N-SIM system operated at a time resolution of 2.3 s (clipped from S2 Movie). In the first image sequence, the cluster split into two separate clusters. In the second sequence, the cluster indicated by the arrowheads translocated along the mesh and merged into the larger cluster. **(B)** Images of actin-pl-clusters visualized by Lifeact-mGFP were obtained using an Olympus SD-OSR system operated at a time resolution of 0.5 s, and were sampled every 5 s (sampled and clipped from S1 Movie). In the first image sequence, the cluster in the center elongated and the cluster in the left-bottom corner underwent merging, elongation, and shrinkage. In the second image sequence, the cluster indicated by the arrowhead in the image at 0 s elongated and spread to form a fork morphology, and then split into two fragments. One of these fragments underwent shrinkage, as indicated by the second arrowhead in the image at 25 s. **(C)** Individual actin-pl-clusters that fit into a square region of 0.6 × 0.6 μm, as observed using Olympus SD-OSR system at a time resolution of 0.5 s, were traced using the single fluorescent-molecule tracking software we previously developed, and their trajectories were superimposed on the SRM image (top-right region in S1 Movie). **(D)** The trajectories in the yellow regions in **C** were magnified by a factor of 3.3 and were color-coded into different colors every 5 s (in the order of purple, blue, green, orange, red, and then back to purple). The black and white arrowheads indicate the start and end positions, respectively.

Most of the actin-pl-clusters exhibited continuous dynamic motion and morphological changes on and along the fine actin meshwork (Fig 4A and 4B). In these images, it is clear that the actin-pl-clusters linked two or more actin filaments in the fine actin meshwork, and thus acted as nodes in the actin meshwork. They exhibited five typical dynamic processes on and along the fine actin filament meshwork, sometimes leading to the actin meshwork formation, in the SRM image sequences in the time scale of 1 s to a few tens of seconds (Fig 4A and 4B, S1 and S2 Movies). First, the actin-pl-clusters were typically elongated, often forming a fork-like morphology and sometimes splitting into two clusters. Second, they moved along the actin meshwork. Third, when two clusters encountered each other, they often merged. Fourth, the elongated actin-pl-clusters sometimes exhibited shrinkage. Fifth, they extended from the existing actin meshwork, leading the growth of an actin filament and the connection of an existing actin meshwork. Since the movements of the actin-pl-clusters or nodes include such complex processes, it is likely that these dynamic processes do not simply represent movements, but might show/include the results of fast actin polymerization-depolymerization processes that occur at the actin-pl-clusters.

These movements were roughly quantitated in the following way. Approximately 85% of the actin-pl-clusters found in the SRM images (4,419 clusters out of 5,199 clusters), such as those shown in Fig 2A, could be fit into a square region of 0.6 × 0.6 μm (15 × 15 pixels; 40 x 40 nm/pixel), and so the center of the two-dimensional signal intensity distribution for each cluster (of square sizes less than 0.60 μm) was determined. The movement of each center was traced, using the single fluorescent-molecule tracking software we previously developed. Since the xy-resolutions of the SRM employed here (an Olympus SD-OSR system) were ~120 nm at the mGFP emission wavelength of 509 nm, the actual diameters of these smaller actin-pl-clusters would be less than 360 nm (see further results in the next subsection).

These clusters could often be tracked for a few tens of seconds (Fig 4C and 4D). Almost all of the actin-pl-clusters appeared to move along fine actin filament meshes parallel to the PM, within a thickness of ~400 nm, which is the axial spatial resolution of the SRMs employed in this study (for an example, see the top-right region in S1 Movie).

### A large majority (91%) of actin-pl-clusters are located within 100 nm from the bottom PM, and visible by total internal reflection fluorescence microscopy (TIRFM)

**Fig 5:**
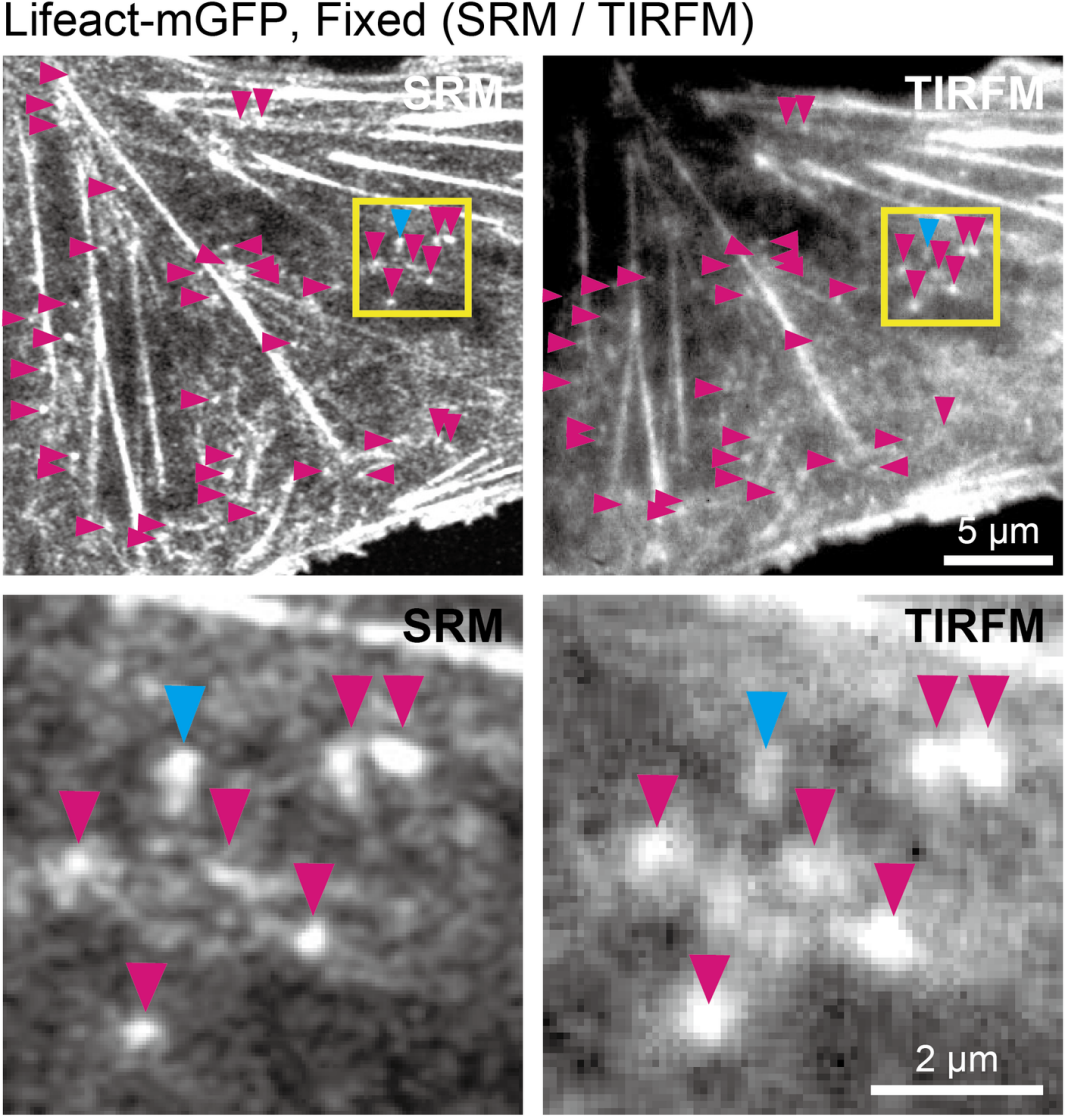
Comparison of actin-pl-cluster images observed by TIRFM with those by SRM. **(Top)** Observations of NRK cells expressing Lifeact-mGFP after fixation was performed in the same field of view, first **(Top-left)** with a Zeiss Airyscan SR microscope (38 nm/pixel) and then **(Top-right)** with our custom-built TIRF microscope based on a Nikon Ti-E inverted microscope (68 nm/pixel). Arrowheads indicate the actin-pl-clusters observed in each image. **(Bottom)** The regions within the yellow boxes in the top images are magnified in the bottom images. The magenta arrowheads indicate the actin-pl-clusters observed in both the SRM and TIRFM images and the cyan arrowheads indicate an actin-pl-cluster that could be observed clearly in the SRM image, but was blurred in the TIRFM image.

We examined the behaviors of actin-pl-clusters on and near the ventral (basal) PM cytoplasmic surface using total internal reflection fluorescence microscopy (TIRFM), which typically allows for the detection of actin-pl-clusters located within 100 nm from the coverslip. Using the cells expressing Lifeact-mGFP after fixation, SRM images and TIRFM images of the same field of view were compared. The fixed cell specimens were first observed with a Zeiss Airyscan SR microscope (~400-nm axial resolution) and then with our custom-built TIRF microscope based on a Nikon Ti-E inverted microscope, which can visualize fluorescent molecules located within ~100 nm from the top surface of the glass coverslip (Fig 5 top row).

As shown in Fig 5 (top panels), many stress fibers found in the SRM image were visible in the TIRFM image. However, the fine meshwork structures seen in the SRM image were blurred and lost in the TIRFM image. This lack of resolution was the reason why studies of the actin meshwork structures located near the PM in living cells could not be performed until the advent of SRM.

The actin-pl-clusters detected in the SRM images were often also found in the TIRFM images (see the arrowheads in Fig 5 top and bottom panels. Images without the arrowheads are shown in S2 Fig). Image quantification revealed that 91% of the actin-pl-clusters found in the SRM images could also be identified in the TIRFM images, indicating that a majority of the actin-pl-clusters are located within 100 nm from the ventral PM cytoplasmic surface. Although in the previous subsection we described that the actin-pl-cluster diameters are mostly less than 360 nm, the results of the TIRFM observations, combined with the 3D electron tomography results described in the Introduction [6,7], suggested that their diameters are generally much less than 100 nm.

### Actin-pl-clusters with diameters smaller than ~0.6 μm in TIRFM images undergo myosin II-dependent directed diffusion near the PM

**Fig 6:**
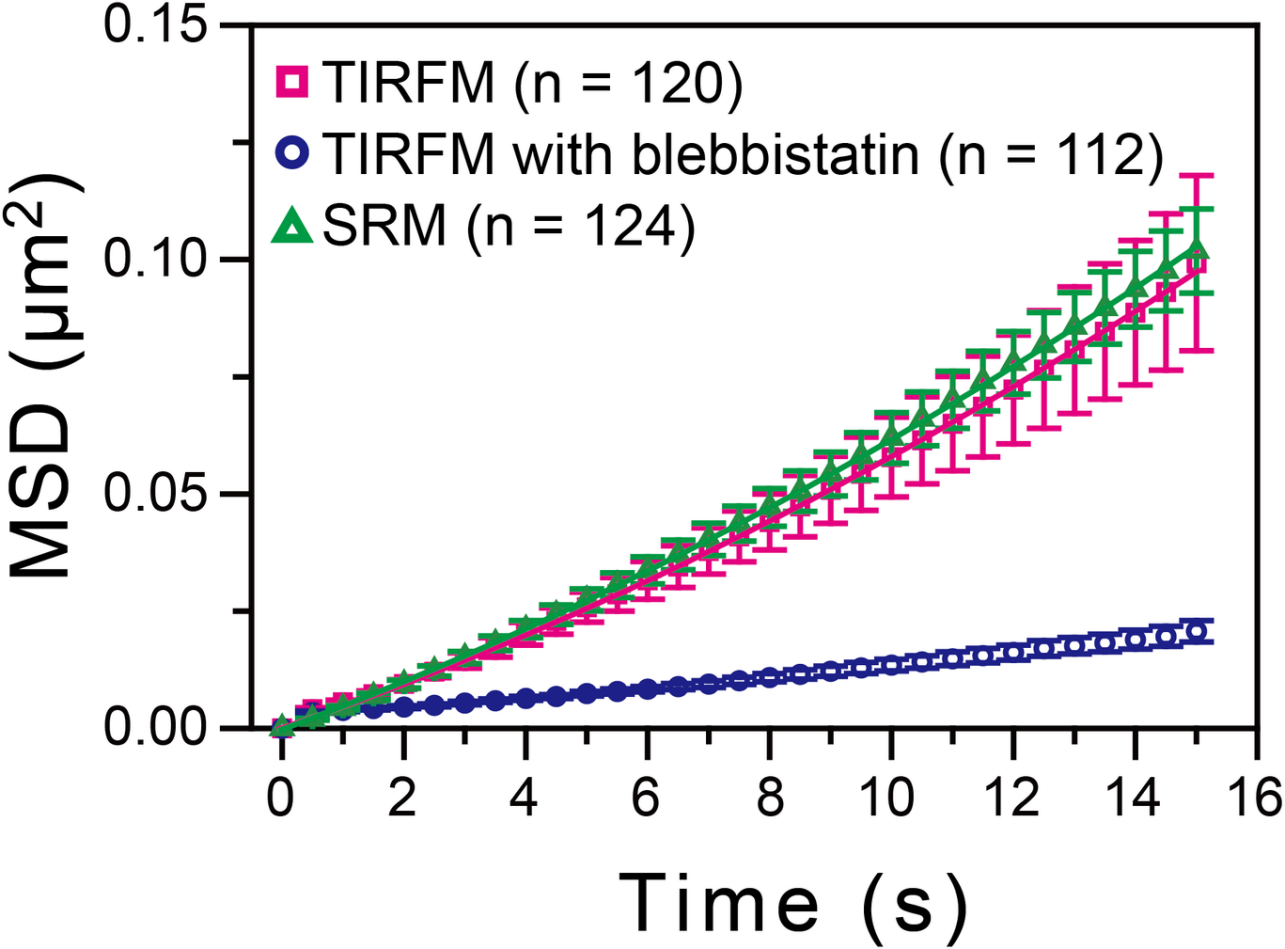
The actin-pl-clusters observed using SRM and TIRFM exhibited very similar dynamics, which was greatly suppressed by the myosin inhibitor blebbistatin. The trajectories of individual actin-pl-clusters that fit into a square region of 0.6 × 0.6 μm were obtained using SRM (Olympus SD-OSR) and TIRFM at a frame rate of 2 Hz (the actual integration times were 0.5 s and 0.167 s, respectively). The ensemble-averaged MSD-*Δt* plots (with error bars indicating standard errors) were obtained for the actin-pl-clusters observed by SRM (green triangles; 124 trajectories in five cells), TIRFM (purple squares; 120 trajectories in five cells), and TIRFM after the treatment with 25 μM blebbistatin, an inhibitor of myosin II (blue circles; 112 trajectories in five cells). The plots for SRM and TIRFM (before blebbistatin addition) were fitted by the equation MSD(*Δt*) = 4*DΔt* + (*vΔt*)^2^ representing the model of Brownian diffusion + directed motion, where *D* is the diffusion coefficient and *v* is the drift velocity. The fitting of the SRM data yielded the dark green curve with *v* = 0.011 ± 0.00016 μm/s and *D* = 0.0013 ± 0.000014 μm^2^/s and the fitting of the TIRFM data yielded the dark magenta curve with *v* = 0.013 ± 0.00019 μm/s and *D* = 0.0010 ± 0.000018 μm^2^/s (the error bars indicate the 68.3% confidence limit of the fitting).

We quantitatively examined the dynamic behaviors of the actin-pl-clusters observed using TIRFM and SRM. For the SRM observations, an Olympus SD-OSR was employed because of its fast observation frame rate of 2 Hz (the actual integration time was 0.5 s). To perform the same analysis, time-lapse TIRFM observations were conducted at the same rate. For the quantitative analysis, we selected actin-pl-clusters with image diameters less than 600 nm for both TIRFM and SRM, and thus the selected actin-pl-clusters were likely to have diameters less than 360 nm in the TIRFM analysis (owing to the spatial resolution of ~240 nm) and less than 480 nm in the SRM analysis (owing to the spatial resolution of ~120 nm). The movements of these actin-pl-clusters were analyzed by using the mean-squared displacement (MSD) averaged over all observed actin-pl-clusters, plotted as a function of the time interval (*Δt*) (Fig 6). The ensemble-averaged MSD-*Δt* plot was fitted by the equation based on the model of Brownian diffusion + directed motion (drift motion):

$$MSD(\Delta t)=4D\Delta t+{\left(v\Delta t\right)}^{2}$$

where *D* is the diffusion coefficient and *v* is the drift velocity. If the motion of actin-pl-clusters is completely random, then *v* will be 0. The fitting results indicated that the ensemble-averaged MSD-*Δt* plots for both the TIRFM and SRM results could be far better fitted (based on both Akaike and Bayesian information criteria) with a non-zero drift velocity (see the caption to Fig 6 for actual values). Furthermore, the Steel-Dwass multiple comparison test indicated no statistically significant difference in the ensemble-averaged MSD-*Δt* plots between the TIRFM and SRM results (*P* = 0.95). This result, in addition to the data shown in Fig 5 (91% of the actin-pl-clusters found in the SRM image could be identified in the TIRFM image), further indicates that the behaviors of actin-pl-clusters could be analyzed using TIRFM.

After treating the cells for 5 min with 25 μM blebbistatin, an inhibitor of myosin II, the actin-pl-clusters were immobilized. Quantitative analysis using the ensemble-averaged MSD-*Δt* plot indicated strong suppression of both the diffusion and drift motions of the actin-pl-clusters (observed by TIRFM; Fig 6; *P* = 2.6 × 10^−6^ for comparison between before and after blebbistatin addition using the Steel-Dwass multiple comparison test). This result suggests that the myosin II activity was responsible for both the fluctuating (apparently diffusing) motion and the directed translocation of actin-pl-clusters (*i*.*e*., both motions might occur as a result of the activity of several myosin filaments that undergo a “tug-of-war,” pulling actin filaments linked to actin-pl-clusters [43]).

### Lifeact conjugated to a transmembrane sequence identified actin structures located within 3.5 nm from the PM cytoplasmic surface

**Fig 7:**
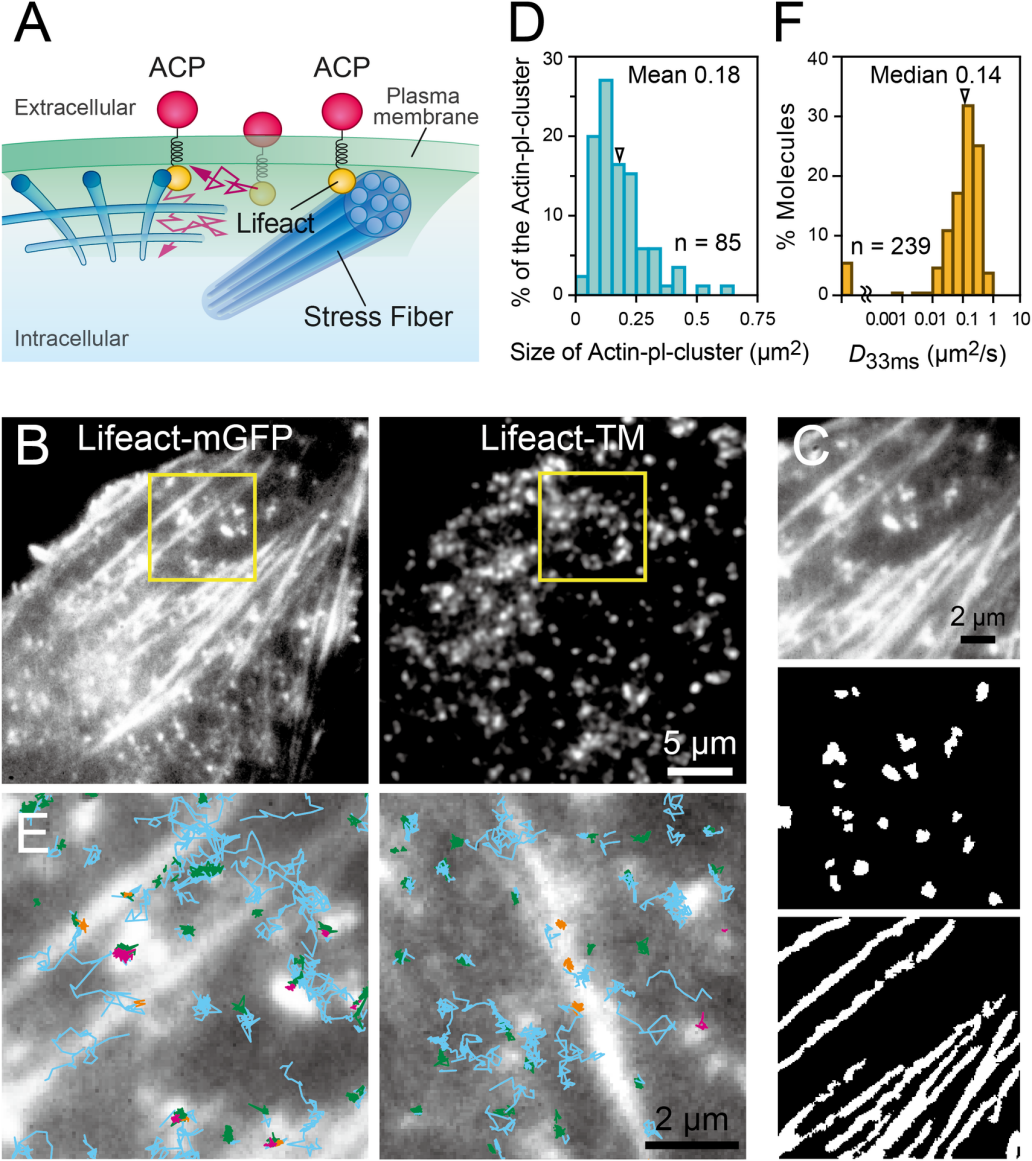
A newly developed probe, Lifeact-TM, detected actin-pl-clusters closely apposed to the ventral membrane. **(A)** Schematic diagram of a newly developed probe for the detection of actin-concentrated structures (including mesh-like structures, stress fibers, and actin-pl-clusters) in close proximity to the PM cytoplasmic surface, named Lifeact-TM, with an ACP tag for fluorescence labeling as the extracellular domain. **(B)** Snapshot images from two-color TIRFM observations of **(Left)** Lifeact-mGFP to visualize actin-pl-clusters and **(Right)** single Lifeact-TM molecules labeled with SeTau647. **(C) (Top)** The region within the yellow square of the Lifeact-mGFP image shown in **B Left**. The image was binarized to optimally extract **(Middle)** actin-pl-clusters and **(Bottom)** stress fibers. **(D)** The area size distribution of binarized actin-pl-clusters (85 clusters in five cells). The arrowhead indicates the mean value. **(E) (Left)** The single-molecule trajectories of Lifeact-TM obtained at 60 Hz (16.7-ms time resolution) are superimposed on the Lifeact-mGFP image shown in **C Top**. Temporary immobilizations, or TALL events, were detected within each trajectory (see the TALL Detection section in the Materials and Methods), and were color-coded based on their mobility and location: blue indicating the mobile period, and magenta, yellow, and green indicating the TALL periods that occurred in the regions of actin-pl-clusters, stress fibers, and elsewhere, respectively. For the original image sequence, see S3 Movie. **(Right)** Another representative TALL analysis result in a different cell. **(F)** The distribution of the diffusion coefficients on the time scale of 16.7–50 ms (*D*_33ms_) of Lifeact-TM during the mobile period (239 trajectories in five cells). The arrowhead indicates the median value.

We examined whether the actin-pl-clusters are closely apposed to the bottom PM cytoplasmic surface. For this purpose, we developed a new fluorescent probe in which Lifeact is linked to the N-terminus of a type II single-pass PM transmembrane (TM) protein, syntaxin 4 (1‒37 amino acids in the N-terminus cytoplasmic domain + the TM domain [a.a. 267‒298]), using a 15 amino-acid random-coil sequence linker (3 × SGGGG with an expected average diameter of 1.36 nm [44]) and to an acyl carrier protein (ACP) tag as the C-terminal extracellular domain for fluorescence labeling (Lifeact-TM; Fig 7A). Assuming that the N-terminal domain of syntaxin 4 forms a random coil, as is the case with the N-terminal 1‒28 amino acid sequence of the related protein syntaxin 1a [45,46], the average diameter of the N-terminal domain of syntaxin 4 would be 2.13 nm [44]. Therefore, in this design, the Lifeact sequence (17 amino acids) is likely to be located approximately 3.5 nm away from the PM cytoplasmic surface (if the syntaxin cytoplasmic domain and the linker together form a random coil, the most likely distance of the Lifeact from the PM cytoplasmic surface would be approximately 2.5 nm), although the distance might vary over time because of the nature of the random coil structure. Lifeact-TM should undergo translational diffusion in the PM, and if Lifeact-TM becomes immobilized, then this would suggest that it became bound to actin filaments located very close (within 3.5 nm) to the PM cytoplasmic surface.

Cells were transfected with cDNAs encoding both Lifeact-mGFP and Lifeact-TM, and Lifeact-TM expressed on the cell surface was labeled with a non-permeable fluorescent dye, SeTau647. Lifeact-TM is continuously internalized, and therefore all of the observations of Lifeact-TM (labeled with SeTau647) were conducted within 10 min after the addition of the SeTau647 probe. Lifeact-mGFP and Lifeact-TM (ACP-SeTau647) were simultaneously observed in two colors at 60 Hz (16.7-ms time resolution), using TIRFM at the single-molecule level for Lifeact-TM and at the multiple-molecule level for Lifeact-mGFP (typical images are shown in Fig 7B).

Under these observation conditions using TIRFM, stress fibers and actin-pl-clusters were clearly visible, but the fine actin meshwork was not, as explained in Fig 5 (Fig 7B, left). Such Lifeact-mGFP images were binarized under the conditions where the regions of either actin-pl-clusters or stress fibers are optimally extracted, and by visual comparison of the two images, the binarized images of actin-pl-clusters and stress fibers were produced (making them complementary to each other; when an overlap of the actin-pl-clusters and stress fibers appeared in the original image [8-bit images], since the stress fibers generally exhibited stronger signals, the overlapped sites were treated as being part of the stress fibers) (Fig 7C). Therefore, in the remaining part of this report, we only consider the actin-pl-clusters that are not entirely masked by the presence of stress fibers in the image.

The distribution of the area sizes of the binarized images indicating actin-pl-clusters is shown in Fig 7D, showing a mean (median) area size of 0.18 ± 0.012 (0.15) μm^2^ (n = 85 actin-pl-clusters; n = 5 cells), and indicating a mean diameter of 480 nm when the average shape of the cluster is approximated to be a circle. This result suggests that the actual mean diameter of the actin-pl-clusters would be ~240 nm (owing to the spatial resolution of ~240 nm of TIRFM).

To examine the possibility that Lifeact-Halo (TMR conjugated) and Lifeact-TM affect the actin dynamics and organization, particularly near the bottom PM cytoplasmic surface, the calponin homology domain of utrophin fused to EGFP at the N-terminus (GFP-UtrCH), another known actin probe [47], was expressed in NRK cells and observed by TIRFM (S3 Fig, top). The observed cortical actin structures were very similar to those detected with Lifeact-mGFP (Fig 5 right panels and Fig 7B). When NRK cells were cotransfected with GFP-UtrCH together with Lifeact-Halo or Lifeact-TM, the stress fibers and actin-pl-clusters visualized by GFP-UtrCH appeared to be similar to those observed without cotransfection with Lifeact probes (S3 Fig, the middle and bottom rows), suggesting the minimal effect of Lifeact probes on the cortical actin organization under the conditions employed here.

Single Lifeact-TM molecules were clearly visible (Fig 7B, right) and exhibited rapid translational diffusion in the PM, with intermittent immobilizations (S3 Movie), which are behaviors that we previously referred to as Temporary Arrest of LateraL diffusion (TALLs; this word is derived from the term STALL, which we originally coined as Stimulation-induced Temporary Arrest of LateraL diffusion [38], but when temporary cessation of lateral diffusion occurs without stimulation-induction, we call it TALL [48,49]). Fig 7E shows instances where the images of Lifeact-TM, which are shown in Fig 7C, are superimposed onto the trajectories of Lifeact-TM spots, which are shown in the right inset of Fig 7B. The trajectories of Lifeact-TM are color-coded based on their mobility and the location: blue indicates the mobile period, and magenta, yellow, and green indicate the TALL periods that occurred in the regions of actin-pl-clusters, stress fibers, and elsewhere (this is expected to occur when the molecule binds to the actin meshwork or actin-based membrane skeleton, although it is not detectable using TIRFM), respectively. Among all of the TALLs observed in the present study, 13% occurred on actin-pl-clusters, 21% on stress fibers, and 66% on the actin-based membrane skeleton (Table 1). The diffusion coefficient of Lifeact-TM during the mobile period (median = 0.14 μm^2^/s; Fig 7F) was slower as compared with those of other single-pass TM proteins, such as Halo-TM (0.26 μm^2^/s [48]), suggesting the possibility that Lifeact-TM undergoes frequent but much shorter TALLs, which are undetectable with the present instrumentation settings.

**Table 1:** The percentage of TALL events that Lifeact-TM molecules exhibited on actin-pl-clusters, stress fibers, and the cortical actin meshwork (the PM regions on which these structures are projected), and the percentage of at least one TALL event once Lifeact-TM entered the PM region on the three actin structures (the projected PM regions).

PM region	%TALLs occurred (Mean ± SE)	%TALLs once Lifeact-TM entered the PM region on the actin structure (Mean ± SE)	Total # of TALLs	Total # of structures	Total # of cells examined
Actin-pl-clusters	12.8 ± 2.2	71.5 ± 4.3	116	94	5
Stress fibers	20.8 ± 6.9	89.4 ± 4.8	196	46	4*
Cortical meshwork	66.4 ± 6.8	N.A.^†^	628	N.A.*	5

*One of the five cells inspected did not show the presence of stress fibers, and therefore, the number of cells examined is one less than those of the other structures.

^†^Not applicable because the regions covered by the cortical actin meshwork are often well connected and represent very high percentages of the PM area, making the evaluation meaningless (~100% for almost all of the cells).

### More than two-thirds of actin-pl-clusters are closely apposed to the bottom PM cytoplasmic surface

Next, we estimated how many actin-pl-clusters and stress fibers were located within the distance where Lifeact-TM can bind to them (<3.5 nm from the basal PM cytoplasmic surface). For this purpose, we first identified the Lifeact-TM molecules entering the PM region, onto which the images of actin-pl-clusters and stress fibers were projected, and stayed there for at least five frames (83.5 ms for an observation time resolution of 16.7 ms). We then examined whether these molecules underwent at least one TALL event in the projected region (on the PM) of actin-pl-clusters and stress fibers for at least 0.25 s (15 frames at a 16.7-ms resolution). When a TALL event of Lifeact-TM occurred in the projected region, it was interpreted as evidence that the projected region is located within 3.5 nm from the PM cytoplasmic surface. Then, the percentages of the projected PM regions of actin-pl-clusters (stress fibers) that contained the TALL events against those that contained the Lifeact-TM trajectories were determined. This should reliably represent the fraction of actin-pl-clusters (stress fibers) that are apposed to (located within 3.5 nm from) the PM cytoplasmic surface.

The results indicated that 72% and 89% of Lifeact-TM molecules that entered the projected areas of actin-pl-clusters and stress fibers, respectively, underwent at least one TALL event there (Table 1), suggesting that at least 72% of the actin-pl-clusters and 89% of the stress fibers visible by TIRFM are closely apposed to the PM cytoplasmic surface (within a distance of 3.5 nm). Since 91% of the actin-pl-clusters visible by SRM were also found in TIRFM images, we concluded that at least 66% of the actin-pl-clusters are located very close to the PM cytoplasmic surface.

The projected region of a single stress fiber was often colocalized with more than one site of the Lifeact-TM’s TALL event. Since the stress fibers are approximately straight in the scale of a cell (persistence length >> cell size), this result suggests that almost all of the stress fibers visible by TIRFM are located on the PM cytoplasmic surface.

### Myosin and filamin A are not colocalized with or recruited to actin-pl-clusters

The elasticity of an active actin filament network can be controlled by bipolar filaments of myosin II, but for this process to occur, the actin filaments must be crosslinked by filamin A [50]. For the actin meshwork to form asters and to exert or resist force, the myosin II activity is essential [1,51]. Therefore, we next investigated whether filamin A and myosin IIA (detected by labeling myosin regulatory light chain [MRLC]) are located at the actin-pl-clusters or distant from the clusters in the live-cell environment.

Cells were cotransfected with the cDNA encoding Lifeact-mGFP for actin-pl-cluster visualization, together with the cDNA encoding filamin A (tagged with the Halo-Tag protein at its N-terminus, termed Halo-filamin A). Halo-filamin A was labeled with a tetramethylrhodamine (TMR)-linked Halo ligand, and it was visualized simultaneously with Lifeact-mGFP. Meanwhile, to simultaneously observe MRLC and actin-pl-clusters, EGFP fused to the N-terminus of MRLC, (EGFP-MRLC) and Lifeact-Halo were expressed in the cytoplasm, and Lifeact-Halo was labeled with a TMR-Halo-ligand. mGFP and TMR were observed using TIRFM, and the excitation laser intensity and the camera gain for observing Halo-filamin A and EGFP-MRLC were adjusted so that their monomers (single molecules) and clusters of up to five molecules could be visualized within the dynamic range of the camera.

**Fig 8:**
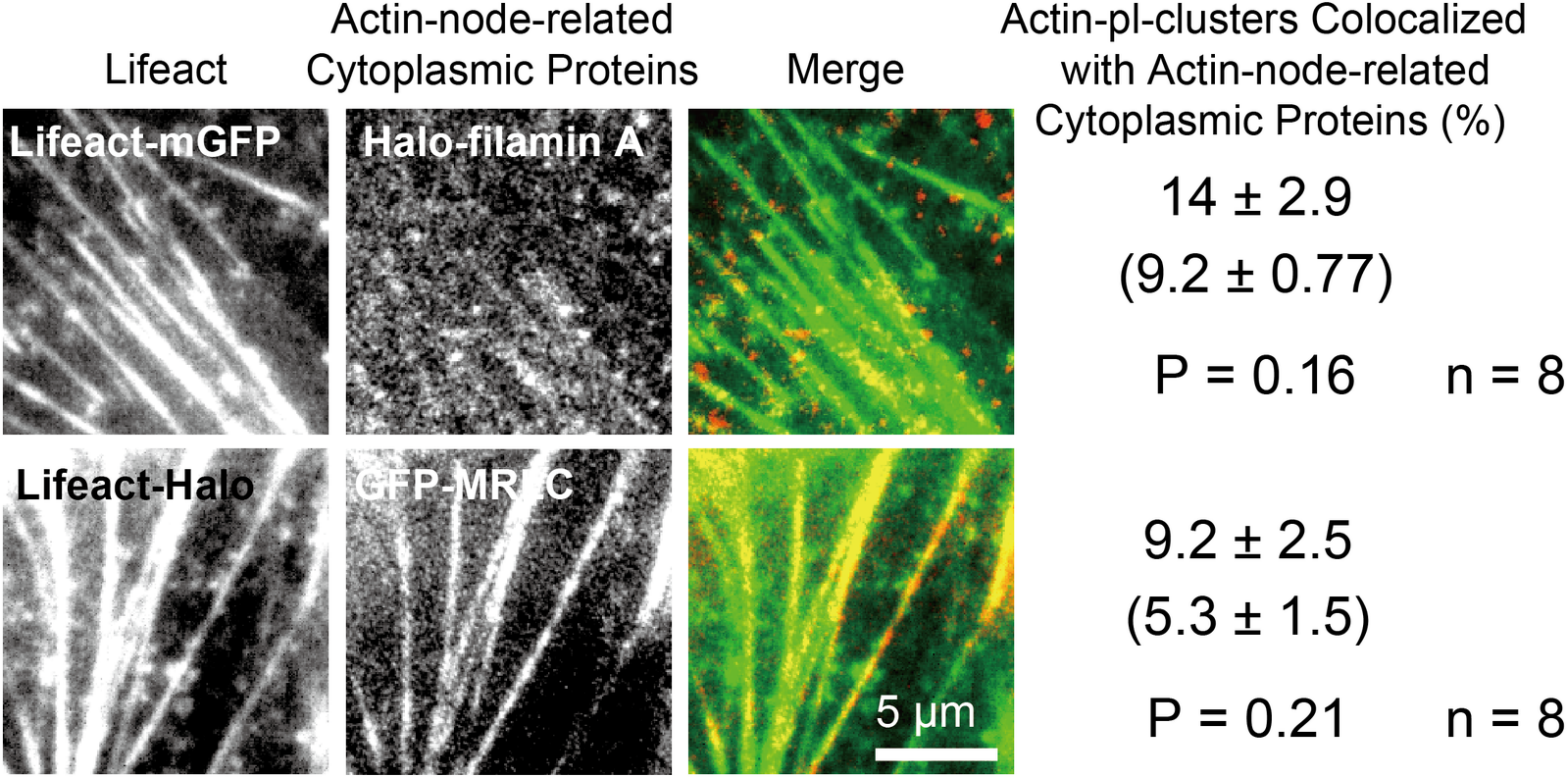
Myosin II and filamin A, the components of actin nodes, did not colocalize with actin-pl-clusters. Two-color TIRFM observations of **(Top row)** Lifeact-mGFP (green) and Halo-filamin A labeled with TMR (red), and **(Second row)** Lifeact-Halo labeled with TMR (green) and EGFP-MRLC (red) were performed at 60 Hz (16.7-ms time resolution), so the monomers of Halo-filamin A and EGFP-MRLC and their clusters of up to five molecules could be visualized within the dynamic range of the camera. In the rightmost column, the top values indicate the percentages of actin-pl-clusters that colocalized with actin-node-related cytoplasmic proteins (Halo-filamin A and EGFP-MRLC). The values in the brackets were obtained as controls to evaluate incidental colocalization, indicating the colocalization percentages when the images of Lifeact-mGFP were rotated 180 degrees. The bottom values are the *P* values of a Student t-test for the colocalization percentages between the correct superimpositions and the rotated superimpositions and the number of cells examined. No statistically significant colocalizations were found for these actin-node-related cytoplasmic proteins.

As shown in Fig 8, the TIRFM Lifeact-mGFP and Lifeact-Halo (TMR) images exhibited the presence of both stress fibers and actin-pl-clusters, as shown in Figs 5, 7B and 7C. As discussed, we only considered the actin-pl-clusters that are not entirely masked by the presence of stress fibers in the image (although here we did not employ binarization of the images). The TIRF images of Halo-filamin A and GFP-MRLC also exhibited stress-fiber-like structures and punctate structures; the Halo-filamin A images exhibited more punctate patterns than the stress-fiber-like structures, and the GFP-MRLC images showed more stress-fiber-like structures. Since we only considered the actin-pl-clusters that are not totally masked by stress fiber images, in the analyses of Fig 8, we only considered the spots (excluding fibers) in the images of Halo-filamin A and GFP-MRLC.

To avoid the problems of overexpression of Halo-filamin A and EGFP-MRLC, we employed conditions where these proteins are expressed at low levels, and detected at single-molecule levels. The number of Halo-filamin A molecules recruited to the PM cytoplasmic surface was much less as compared with that of EGFP-MRLC, but the expression of Halo-filamin A was confirmed by western blotting (S4 Fig).

A comparison of the image of Lifeact-mGFP (Fig 8, top left) with that of Lifeact-Halo-TMR (Fig 8, second row, left) revealed that both exhibited prominent stress fibers and many punctate actin-pl-clusters. This result suggests that although Lifeact-Halo-TMR has never been used to probe f-actin, it would be as appropriate as Lifeact-mGFP for observing actin filaments and actin-pl-clusters (also see S3 Fig).

First, we examined the percentage of actin-pl-clusters (labeled with Lifeact-mGFP) that colocalized with Halo-filamin A. As a control, an image of Lifeact-mGFP was rotated 180 degrees and the percentage of incidental colocalizations was evaluated. The Student t-test of the colocalization percentages between the correct superimpositions (14 ± 2.9% actin-pl-clusters colocalized by filamin A; n = 8 cells) and rotated superimpositions (9.2 ± 0.77% actin-pl-clusters colocalized by filamin A; n = 8 cells) showed no statistically significant colocalization.

Next, the colocalization of EGFP-MRLC and actin-pl-clusters (labeled with Lifeact-Halo-TMR) was examined in the same manner. The Student t-test for the colocalization percentages between the correct superimpositions (9.2 ± 2.5% actin-pl-clusters colocalized by MRLC; n = 8 cells) and flipped superimpositions (5.3 ± 1.5% actin-pl-clusters colocalized by MRLC; n = 8 cells) showed no statistically significant colocalization.

Therefore, we concluded that filamin A and myosin IIA (MRLC) are not located with the actin-pl-clusters in the live-cell environment, which is at variance with the results obtained in cells treated with latrunculin A [18] and in *in vitro* reconstituted actomyosin-membrane systems [19]. This result might appear to conflict with the strong effect of blebbistatin on the actin-pl-cluster dynamics, shown in Fig 6. However, the results shown in Fig 6 clearly indicate that the myosin II activity was responsible for both the fluctuating motion (apparently diffusing motion) and the directed translocation of the actin-pl-clusters, suggesting that both motions might occur as a result of the activity of several myosin filaments that undergo a “tug-of-war,” pulling actin filaments linked to actin-pl-clusters in several different directions at the same time. The lack of MRLC colocalization with the actin-pl-clusters suggests that the myosin II filaments that participate in a tug-of-war, pulling on an actin-pl-cluster, would be somewhat distant from the actin-pl-clusters. As a result of the fluctuating total force generated by several myosin II filaments located away from the actin-pl-cluster, the actin-pl-cluster would undergo fluctuating motion as well as directed translocation.

### Actin-pl-clusters (actin nodes found here) differ from actin nodes/asters, as revealed by latrunculin treatments

The lack of colocalization of filamin A and MRLC with actin-pl-clusters suggests that the actin-pl-clusters found in this study in intact HeLa cells are probably different from the actin asters found in the reconstituted actomyosin system in the presence of the artificial membrane [19] and the actin nodes/asters found in live HeLa cells after a treatment with 200–800 nM latrunculin A [18]. To further clarify whether the structures of the actin-pl-clusters found here are similar to the actin nodes/asters detected after the latrunculin treatment, we used an SRM (SDSRM; an Olympus SD-OSR system) to observe the actin structures after a 200 nM latrunculin treatment, in both the HeLa cells employed by Luo et al. [18] and the NRK cells used here.

**Fig 9:**
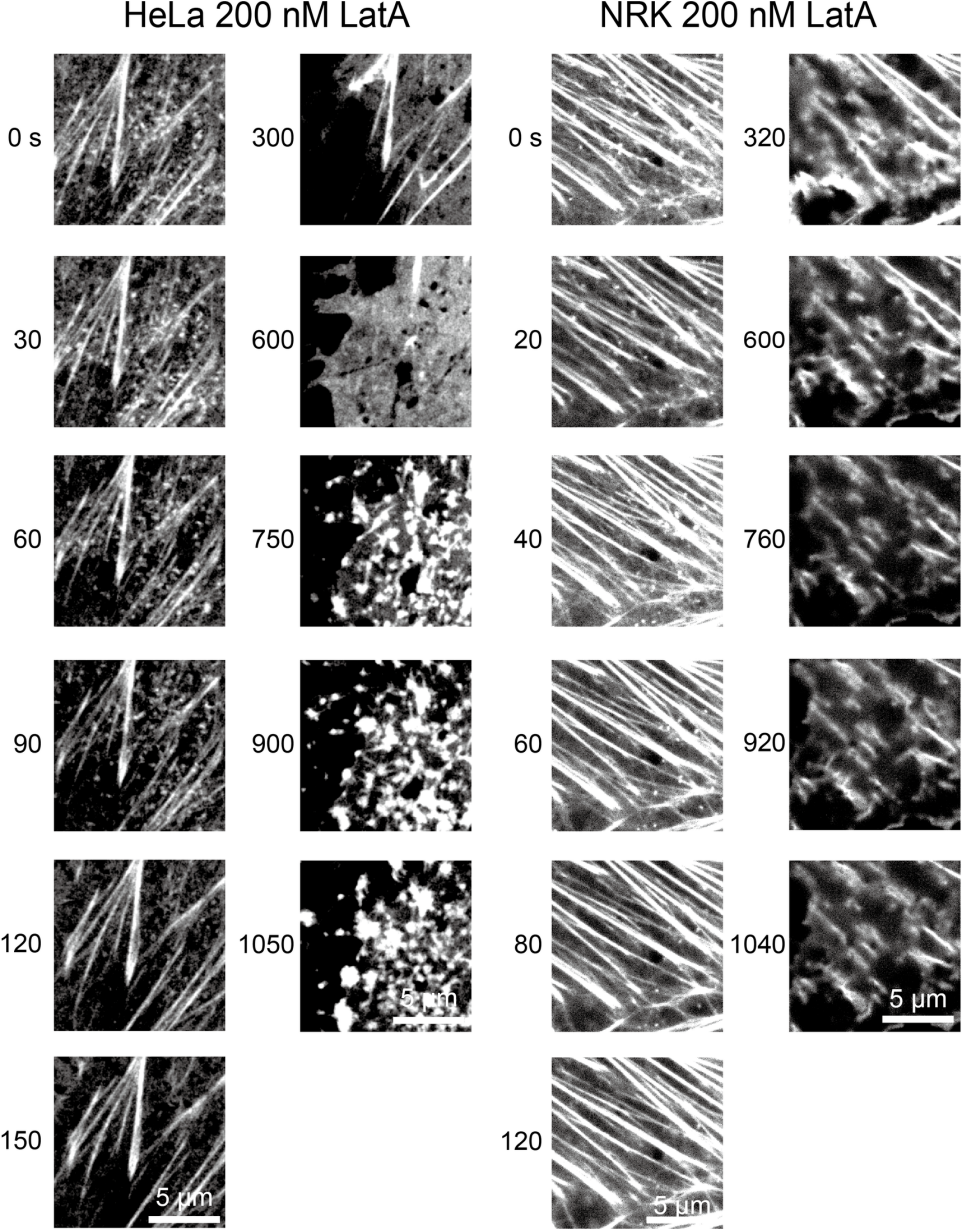
Actin-pl-clusters were found in intact HeLa cells as well as in the NRK cells extensively used in this study: After the addition of 200 nM latrunculin A, actin-pl-clusters disappeared in 1~2 min in both HeLa and NRK cells, but in HeLa cells, actin clusters much larger than the actin-pl-clusters started appearing between 600 and 750 s after latrunculin addition. The figure shows representative snapshots from image sequences of HeLa cells **(Left)** and NRK cells **(Right)** transfected with Lifeact-mGFP and observed by SRM (an Olympus SD-OSR system). After the snapshots at time = 0 (0 s), the cells were treated with 200 nM latrunculin A and time-lapse observations were performed for about 1,000 s.

First, actin-pl-clusters, in addition to stress fibers, were found in intact HeLa cells expressing Lifeact-mGFP, as in NRK cells (Fig 9 top left). The time-dependent changes of the Lifeact-mGFP-bound structures were then observed, after the addition of 200 nM latrunculin A. The actin-pl-clusters disappeared almost entirely in 1~2 min in both HeLa and NRK cells, whereas some stress fibers remained for longer periods, although many fibers disappeared and some broke up into pieces.

Interestingly, in the HeLa cells used by Luo et al., actin clusters much larger than the actin-pl-clusters, which are probably the actin nodes/asters reported by Luo et al., started appearing between 600 and 750 s after the latrunculin addition, followed by further increases in size (accompanied by a reduction in the number of clusters). The appearance of large actin clusters after the disappearance of the actin-pl-clusters is consistent with the results reported by Luo et al., but at variance with their description (particularly, the text referring to Panel E of Fig 1), the actin-pl-clusters that existed before the latrunculin treatment were much smaller than the actin clusters that appeared some time after the latrunculin treatment. Namely, we conclude that these two actin clusters are quite different structures, and probably have distinct molecular compositions.

In the case of the NRK cells employed in this study, although the addition of 200 nM latrunculin A induced the disappearance of the actin-pl-clusters, this was not followed by the appearance of greater actin clusters (at least up to ~1,000 s). These results again suggest that the actin-pl-clusters (actin nodes found here) are probably different from the actin nodes/asters that appeared after latrunculin treatment, reported by Luo et al.

### Podosome-related cytoplasmic proteins are transiently recruited to actin-pl-clusters, one molecule after another

**Fig 10:**
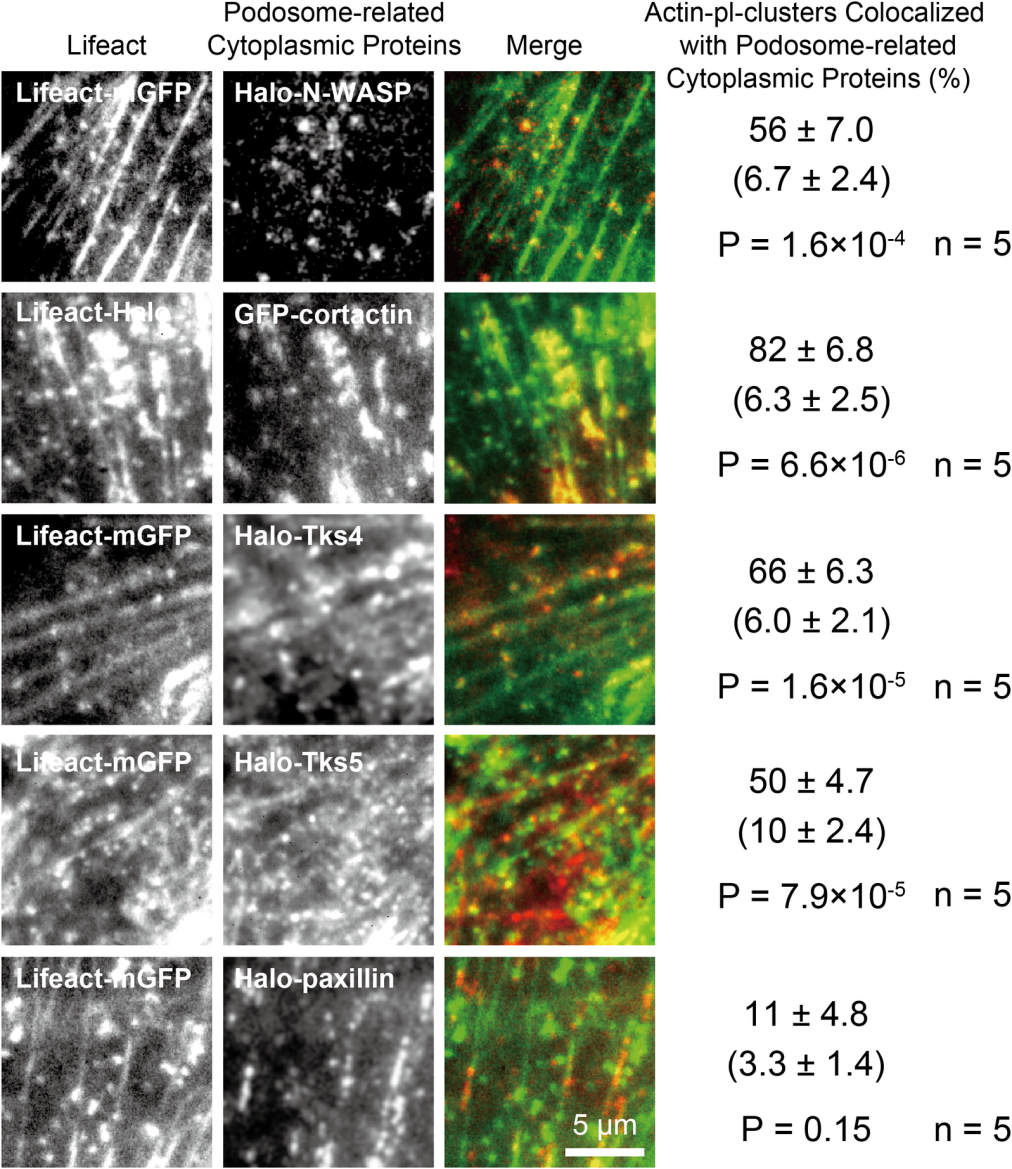
Podosome-related proteins N-WASP, cortactin, Tks4, and Tks5 colocalized with actin-pl-clusters. Two-color TIRFM observations of **(Top row)** Lifeact-mGFP (green) and Halo-N-WASP labeled with TMR (red) (see S4 Movie), **(Second row)** Lifeact-Halo labeled with TMR (green) and EGFP-cortactin (red), **(Third row)** Lifeact-mGFP (green) and Halo-Tks4 labeled with TMR (red) (see S5 Movie), **(Fourth row)** Lifeact-mGFP (green) and Halo-Tks5 labeled with TMR (red) (see S6 Movie) and **(Bottom row)** Lifeact-mGFP (green) and Halo-paxillin labeled with TMR. The observation conditions and the analyses were the same as in Fig 8. Statistically significant colocalizations with actin-pl-clusters were found for N-WASP, cortactin, Tks4, and Tks5.

The spatial distribution of the actin-pl-clusters was reminiscent of that of podosomal proteins (for examples, see Figs 3 and 4 in Kaksonen et al. [52] and Fig 3 in Stölting et al. [53]; we are grateful to Dr. John Heuser for pointing out this resemblance). Therefore, we examined whether four key podosome-related cytoplasmic proteins, the actin regulators neural Wiskott–Aldrich syndrome protein (N-WASP) and cortactin, and the Tyr kinase substrate family adaptor proteins with four SH3 domains (Tks4) and five SH3 domains (Tks5) [54], are recruited to (or colocalized with) the actin-pl-clusters (cortactin and N-WASP are also known to be involved in actin polymerization [55,56]) (Fig 10). Similar to the analysis of the recruitment of filamin A and MRLC to actin-pl-clusters (Fig 8), the evaluation of the recruitment of these proteins was performed at the single-molecule level, with a dynamic range allowing the observation of clusters composed of up to five molecules, and only the punctate images were evaluated, excluding the fiber-like images.

After the subtraction of incidental colocalization, approximately 49, 76, 60, and 40% (percentages for the correct superimposition minus that for the rotated superimposition) of the actin-pl-clusters were found to be colocalized with N-WASP, cortactin, Tks4, and Tks5, respectively (with incidental colocalizations of ~6‒10%; n = 5 cells). The Student t-test showed statistically significant differences in the colocalization between the correct superimposition and rotated superimposition. Additionally, a major focal adhesion (FA) molecule, paxillin, did not significantly colocalize with the actin-pl-clusters, although the involvement of paxillin and other FA component molecules in the formation of podosomes has been reported [57–59].

Importantly, these molecules were transiently recruited to the actin-pl-clusters, and many molecules were continually recruited one after another (S4, S5 and S6 Movies). Individual N-WASP, Tks4, and Tks5 molecules reached the actin-pl-clusters either directly from the cytoplasm (62.1 ± 5.5%, 66.1 ± 4.2%, and 79.1 ± 8.7%, respectively) or through translational diffusion on the PM cytoplasmic surface after landing there from the cytoplasm. When individual N-WASP, Tks4, and Tks5 molecules left the actin-pl-cluster, approximately 30% of the molecules left the PM cytoplasmic surface and directly entered the cytoplasm (31.2 ± 6.2%, 26.9 ± 6.2%, and 18.7 ± 2.8%).

**Fig 11:**
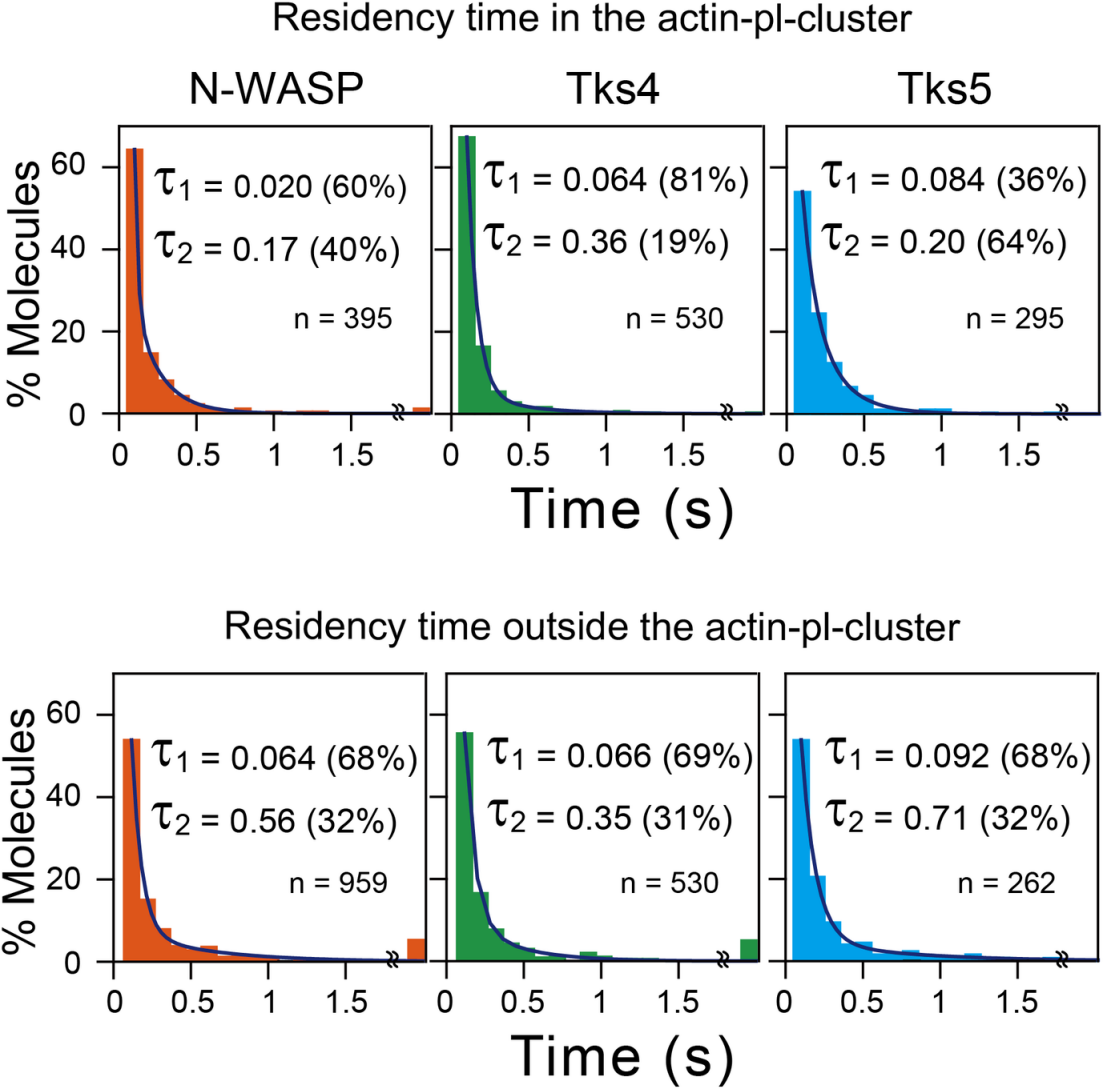
Residency times of N-WASP, Tks4, and Tks5 at the actin-pl-clusters and at the PM outside the actin-pl-clusters. **(Top)** The histograms show the distributions of residency times of individual N-WASP, Tks4, and Tks5 molecules at the actin-pl-clusters. Each distribution could be fitted well by the sum of two exponential functions, yielding the two exponential decay constants with the fraction (%) of each population. The decay constants were then corrected for the photobleaching lifetime of TMR bound to the Halo protein (6.2 s), and the shorter residency time of τ_1_ and the longer residency time of τ_2_ were obtained. For N-WASP, τ_1_ = 0.020 ± 0.0040 (60%) and τ_2_ = 0.17 ± 0.017 (40%), for Tks4, τ_1_ = 0.064 ± 0.0018 (81%) and τ_2_ = 0.36 ± 0.059 (19%) and for Tks5, τ_1_ = 0.084 ± 0.026 (36%) and τ_2_ = 0.20 ± 0.038 (64%). The error bars indicate the 68.3% confidence limit of the fitting, and the numbers of recruitment events (n = 395 for N-WASP, n = 530 for Tks4 and n = 295 for Tks5) were obtained from five cells for each molecule. Since trajectories as short as 1–3 frames (shorter than 50 ms at 60 fps) often include those produced by transient background noise, these short trajectories were excluded from the analysis to avoid overestimation of the number of recruitment events with short residency lifetimes (thus the x-axes of the graphs start from 67 ms). **(Bottom)** The histograms show the distributions of residency times of individual N-WASP, Tks4, and Tks5 molecules at the PM outside the actin-pl-clusters. For N-WASP, τ_1_ = 0.064 ± 0.0098 (68%) and τ_2_ = 0.56 ± 0.22 (32%), for Tks4, τ_1_ = 0.066 ± 0.0037 (69%) and τ_2_ = 0.35 ± 0.044 (31%) and for Tks5, τ_1_ = 0.092 ± 0.0047 (68%) and τ_2_ = 0.71 ± 0.15 (32%). The recruitment events (n = 959 for N-WASP, n = 530 for Tks4 and n = 262 for Tks5) were obtained from five cells for each molecule.

The residency time of each individual molecule in the actin-pl-cluster was measured, and after observing many recruited molecules, histograms of the residency times for N-WASP, Tks4, and Tks5 were obtained (Fig 11; This was not performed for cortactin because regulating its expression levels so that the residency time of each individual molecule could be measured was extremely difficult. Its expression sharply increased from too low to too high for the single-molecule residency time assay.). To correctly determine the residency time of each molecule without the recruitment of another molecule to the same actin-pl-cluster, a Halo-tag labeling efficiency of ~50% was employed for N-WASP, Tks4, and Tks5. The distributions could be well fitted by the sum of two exponential decay functions, as judged by both the Akaike and Bayesian information criteria, providing the lifetimes of 0.020 s (60%) and 0.17 s (40%) for N-WASP, 0.064 s (81%) and 0.36 s (19%) for Tks4, and 0.084 s (36%) and 0.20 s (64%) for Tks5 (all of these lifetimes are after the correction for the TMR photobleaching lifetime of 6.2 s, obtained under the same conditions as those employed for the observations made here). These residency lifetimes in the actin-pl-cluster are quite similar to those outside the actin-pl-cluster (0.064 s [68%] and 0.56 s [32%] for N-WASP, 0.066 s [69%] and 0.35 s [31%] for Tks4, and 0.092 s [68%] and 0.71 s [32%] for Tks5). Perhaps, these molecules bind to other actin-related structures on the PM cytoplasmic surface. In addition, the reason for the presence of the two decay components remains unknown.

### Actin-pl-clusters are dynamically formed and remodeled by constant and fast polymerization and depolymerization occurring simultaneously

**Fig 12:**
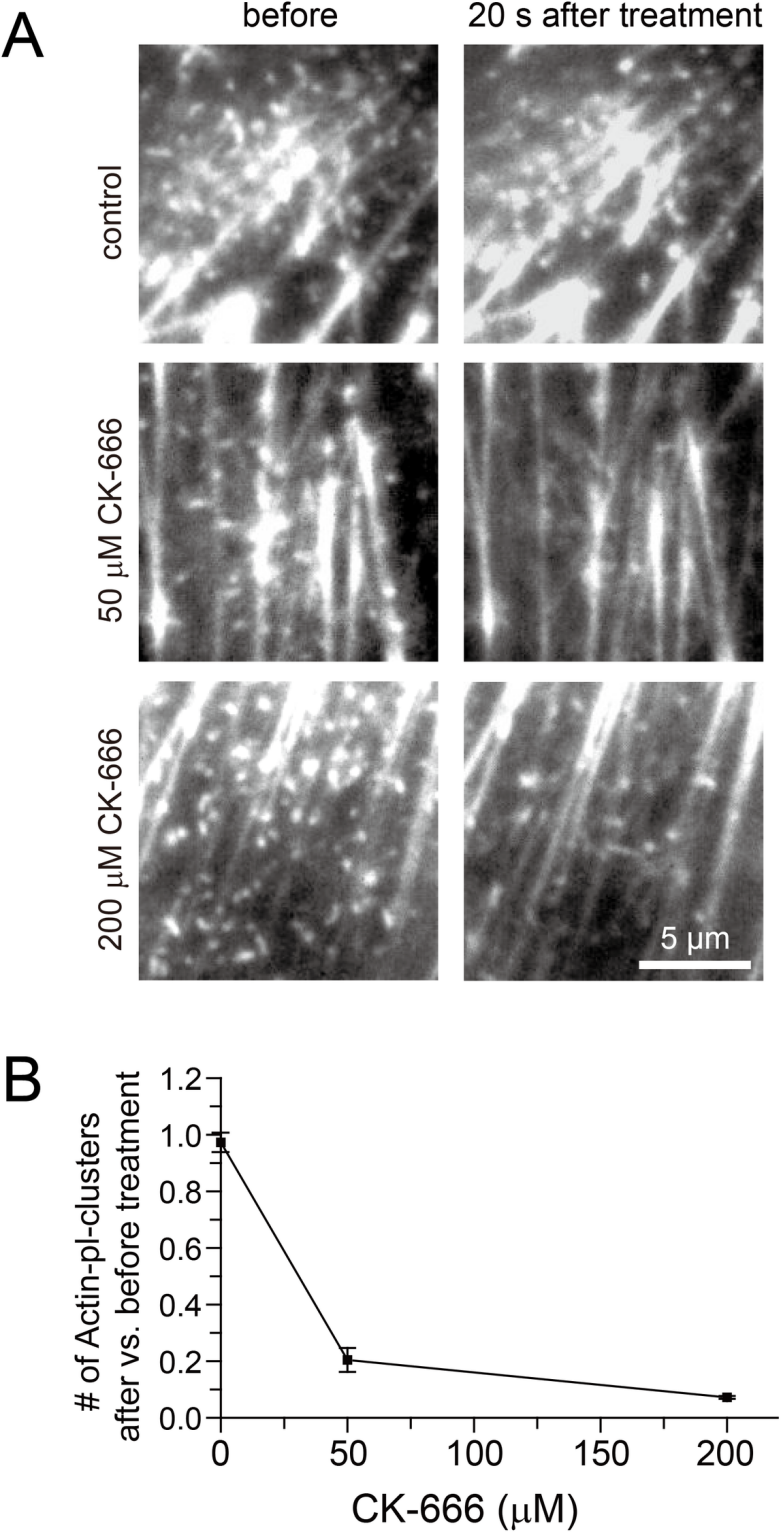
The effect of CK-666, an inhibitor of Arp2/3, on actin-pl-clusters, showing that the number of actin-pl-clusters quickly decreased, *within 20 s*, to the levels of ~20% and ~7.2% of those found before the CK-666 addition. **(A)** Representative snapshots from image sequences of NRK cells transfected with Lifeact-mGFP and observed by TIRFM before **(Left)** and 20 s after the addition of CK-666 **(Right)**. Control, treated only with DMSO. (**B**) The number of actin-pl-clusters observed per cell at 20 s after the CK-666 treatment vs. that before the treatment. The error bars indicate the standard errors (n = 6 cells for each condition).

Arp2/3 are required to initiate the podosome formation, due to their actin nucleation activity. We hoped to observe their colocalizations with (recruitment to) actin-pl-clusters, but we could not obtain sufficient amounts of Arp2 or Apr3 fused to mGFP/Halo expressed on (recruited to) the PM cytoplasmic surface to evaluate their recruitment to the actin-pl-clusters. As another approach, we examined the effect of CK-666, an inhibitor of Arp2/3, on the actin-pl-clusters. Upon the addition of 50 and 200 μM CK-666, the number of actin-pl-clusters quickly decreased, *within 20 s*, to the levels of ~20% and ~7.2% of those found before the CK-666 addition, although actin-pl-clusters with high signal intensity tended to remain (Fig 12). This result is roughly consistent with the IC_50_ of CK-666 (4 and 17 μM for human and bovine Arp2/3, respectively [60]).

This immediate effect of CK-666 was quite surprising, and indicates that the actin nucleation activity of the Arp2/3 complex, which is necessary at the onset of podosome formation [60,61], is also required continuously for maintaining actin-pl-clusters. Namely, this result suggests that actin filaments are continuously polymerized and depolymerized simultaneously at the actin-pl-cluster. Therefore, for the formation and maintenance of the actin-pl-cluster, Arp2/3 complexes would have to be continually recruited to the actin-pl-cluster to induce actin polymerization there.

Considering the continuous transient recruitment of podosome-related proteins, N-WASP, Tks4, and Tks5 (and probably cortactin), to actin-pl-clusters (these molecules are recruited from the cytoplasm to the actin-pl-clusters one after another, and after staying there for a fraction of a second, they depart from the actin-pl-cluster and move into the cytoplasm), the effect of CK-666 clearly shows that the actin-pl-cluster is an extremely dynamic structure, where both polymerization and depolymerization of actin filaments occur continuously by transiently recruiting actin monomers as well as other actin regulating molecules, such as Arp2/3, N-WASP, Tks4, and Tks5. Thus, the actin-pl-cluster constantly remodels its structure and interactions with other actin structures.

## Discussion

Using SRM, combined with TIRFM and single-molecule imaging, we visualized and characterized the actin-pl-clusters. A previous SRM study by Luo et al. [18] found dynamic actin clusters or foci, termed “actin nodes”, but they were mostly identified after partial depolymerization of filamentous actin with latrunculin A. In fact, in this study, we found that the actin-pl-clusters or the actin nodes observed here were quite different structures from those found after the latrunculin treatment (Fig 9). In intact, but mostly fixed cells (without actin depolymerization), using SRMs, such as STORM [2,18], integrating exchangeable single-molecule localization (IRIS; [3]), and advanced SIM [5], the presence of actin nodes or clusters has been seen in published images, but these structures have rarely been mentioned or characterized, even when they were referred to in publications.

In the present study, for the first time, actin-pl-clusters were identified in the cortical actin meshwork by SRM, and their dynamic properties have been extensively characterized (Figs 2 and 4). Furthermore, SRM revealed that virtually all of the actin structures that could be labeled with Lifeact-mGFP, mostly actin-pl-clusters, stress fibers, and the fine actin filament meshwork, were located within 400 nm from the PM cytoplasmic surface (Fig 3), which is important for considering the involvement of actin filaments in various cellular processes. The combined use of SRM with TIRFM was critical for determining that 91% of the actin-pl-clusters were located within 100 nm from the PM cytoplasmic surface (Fig 5) and finding that both the fluctuating/diffusional movements and directed movements of actin-pl-clusters depended on myosin II filaments (Fig 6). By detecting TALLs of Lifeact-TM by single-molecule tracking using TIRFM, 66% of the actin-pl-clusters, 89% of the stress fibers, and some important fractions of the fine actin meshwork were found to be located within 3.5 nm from the PM cytoplasmic surface (Fig 7). By observing single molecules using TIRFM, the transient recruitment of N-WASP, Tks4, and Tks5 (and no recruitment of filamin A and myosin IIA; cortactin was recruited, but its dwell time could not be measured) to the actin-pl-clusters was observed. Namely, the results reported here could only be obtained by the combined use of these advanced methods.

The artifacts of Lifeact conjugated to fluorescent proteins have been reported (for example, see Courtemanche et al. [42]). Therefore, at the initial stages in the present study, the actin structures visualized by Lifeact-mGFP using SRM were comprehensively compared with those stained with Alexa647-phalloidin in fixed cells. The comparison was made for the cells labeled with Alexa647-phalloidin with and without Lifeact-mGFP expression (Fig 1). Similar comparisons using TIRFM were made, by employing GFP-UtrCH-expressing cells (S3 Fig). No effect of Lifeact-mGFP expression on the actin-pl-clusters, cortical actin meshwork, and stress fibers was detected, under the conditions employed here. However, readers should keep in mind that other actin functions in important cellular processes might be affected.

As described in the Introduction, the present research has five clear objectives. In the following, we summarize and discuss the results obtained to address these objectives.

(1) Our SRM studies detected actin-pl-clusters that would act as actin nodes (node-like structures), which link two or more actin filaments in the cortical fine actin meshwork in living cells (Fig 4A and 4B). In fact, this is the first time that the cortical fine actin meshwork (although only a part of it) was directly imaged in living cells (Figs 2 and 4). As described in item 3, the nodes found here differ from the actin nodes/asters previously found under artificial conditions.

(2) Since the actin-pl-clusters were found, we examined 2a) their relationship with other cortical actin structures, 2b) their dynamics and possible causes of their dynamics, and 2c) their locations relative to the PM. With regard to 2a), as shown in Fig 4A and 4B, most of the actin-pl-clusters are located on the cortical actin meshwork, linking two or more actin filaments, and thereby working as nodes for linking actin filaments and enhancing the formation of new filaments.

**Fig 13:**
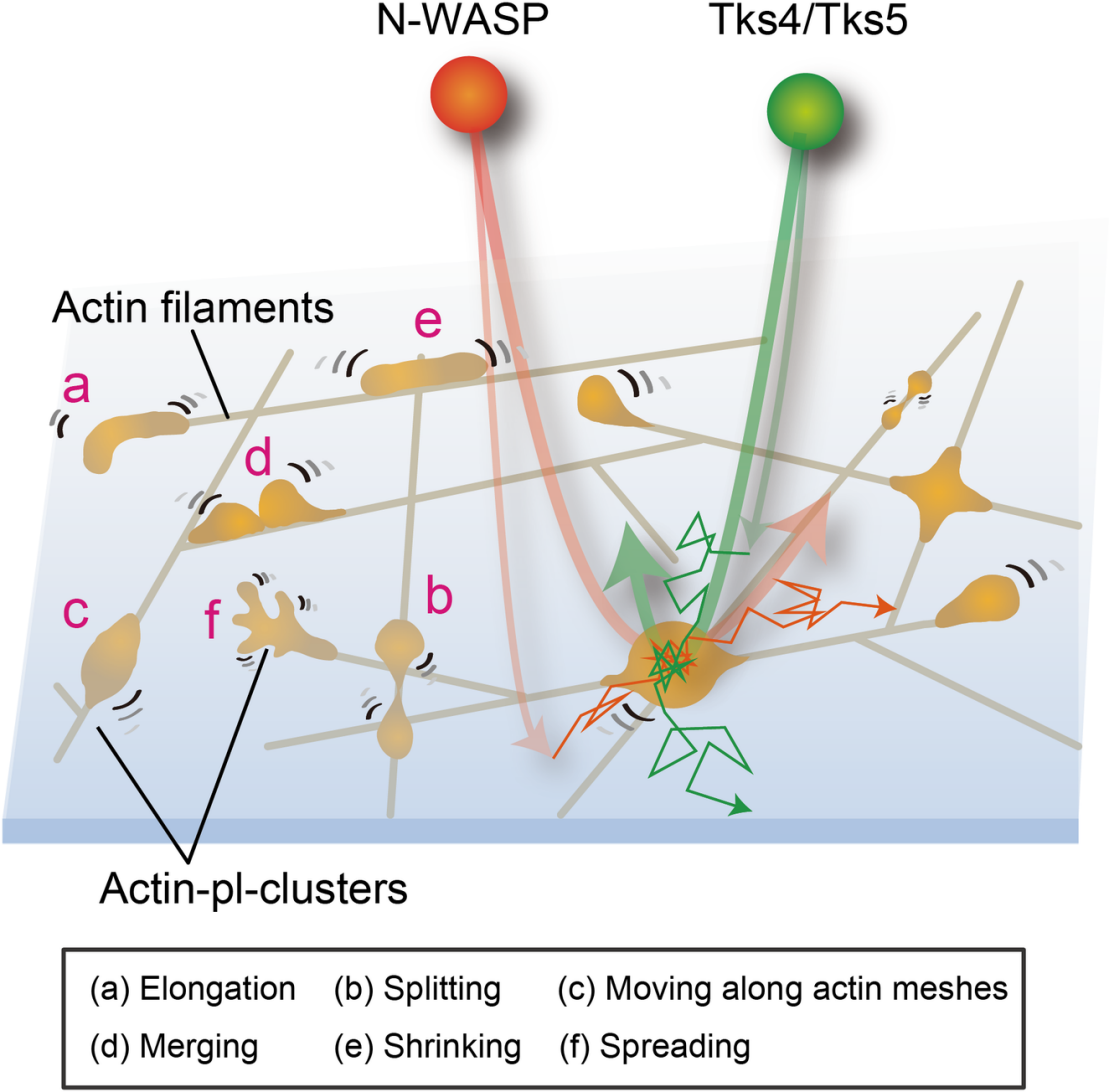
Actin-pl-clusters exhibit dynamic morphological changes and podosome-related proteins are recruited to actin-pl-clusters. Actin-pl-clusters underwent dynamic motion and morphological changes on or near the PM. These include (a) elongation, (b) splitting, (c) directed movement along actin meshes, (d) merging, (e) shrinking, and (f) spreading (often forming a fork-like morphology). N-WASP, Tks4, and Tks5 molecules were either directly recruited to the actin-pl-cluster from the cytoplasm (60–70% of molecules) or first arrived at the PM outside the cluster and were then recruited to the cluster by lateral diffusion on the PM cytoplasmic surface. They left the actin-pl-clusters either by direct dissociation into the cytoplasm (approximately 70% of molecules) or by lateral diffusion on the PM cytoplasmic surface.

For 2b), we found that most of the actin-pl-clusters underwent continuous dynamic motion and morphological changes on and along the cortical fine actin meshwork, generally parallel to the PM, and sometimes leading to the actin meshwork formation (Fig 4A and 4B). As illustrated in Fig 13, the actin-pl-clusters exhibited elongation and shrinkage, with occasional merging and splitting events, while lateral translocation (diffusion + drift) occurred along the cortical actin meshwork, in the time scale of 1 s to a few tens of seconds (Fig 4A and 4B, S1 and S2 Movies). They sometimes extended from the existing actin meshwork, leading the growth of a new actin filament and the connection to an existing actin meshwork.

With regard to the causes of the dynamics, we found that such dynamics occurred in a myosin II-dependent manner. There were two key features of the myosin II function. First, unlike the cases of actin nodes/asters found under artificial conditions, the myosin filaments were located away from the actin-pl-clusters (Fig 8). Second, the inhibition of myosin activity by blebbistatin blocked not only the drift (directed) motion of the actin-pl-clusters on the cortical actin meshwork, but also their fluctuating (diffusion-like) movements (Fig 6). Based on these observations, we propose that several myosin filaments undergo a “tug-of-war” at the actin-pl-cluster, pulling the actin filaments linked to the actin-pl-clusters in several different directions at the same time, and as a result, the actin-pl-cluster undergoes the apparent mixed movements of the fluctuating motion (apparently diffusing motion) and the directed translocation.

Furthermore, some of these movements might be rather apparent ones, representing spatiotemporal variations of the rates of actin polymerization and depolymerization. This is consistent with the recruitment of N-WASP and cortactin to the actin-pl-clusters. In addition, since the CK-666-mediated blocking of the Arp2/3 activity made the actin-pl-cluster disappear on an order of 10 s, rapid continuous and simultaneous polymerization and depolymerization of actin filaments must be occurring at actin-pl-clusters (Fig 12). This might in fact be the mechanism for the formation of actin-pl-clusters.

The rates of actin polymerization and depolymerization at the actin-pl-clusters might be strongly affected by the traction force by the myosin filaments. It is possible that upon blocking the myosin activity, both the actin polymerization and depolymerization might be greatly suppressed, which might in turn suppress the (apparent) movement of the actin-pl-clusters.

The locations of the actin structures relative to the PM (2c) can be summarized in the following way.

1) Virtually all of the actin structures, which are mostly actin-pl-clusters, stress fibers, and the fine actin filament meshwork, are located within 400 nm from the PM cytoplasmic surface (Fig 3).

2) 91% of the actin-pl-clusters are located within 100 nm from the PM cytoplasmic surface (Fig 5).

3) 66% of the actin-pl-clusters, 89% of the stress fibers, and some important fractions of the fine actin meshwork are located within 3.5 nm from the PM cytoplasmic surface (Fig 7).

(3) The actin-pl-clusters, which we found to form nodes in the cortical actin meshwork to link two or more actin filaments, are different from the actin nodes/asters discovered in latrunculin-treated cells by Luo et al. [18], based on the following two observations. 1) Actin-pl-clusters were found in the intact HeLa cells used by Luo et al. as well as in the NRK cells extensively used in this study (Fig 9). After latrunculin treatment, the actin-pl-clusters disappeared almost entirely in 1~2 min in both HeLa and NRK cells. However, only in HeLa cells, actin clusters much larger than the actin-pl-clusters appeared ~500 s later. These larger clusters are likely to be the actin nodes/asters reported by Luo et al. 2) Unlike the actin nodes/asters described by Luo et al., the actin-pl-clusters are not colocalized with either myosin II or filamin A.

(4) Extensive recruitment of N-WASP and cortactin, which are involved in actin polymerization, to actin-pl-clusters was found, consistent with the idea that the actin-pl-clusters function as nodes for linking actin filaments in the meshwork. N-WASP and cortactin are known to colocalize with podosomes. Interestingly, Tks4 and Tks5, which predominantly exist in podosomes, were also colocalized with the actin-pl-clusters. These results suggest that (1) some of the actin-pl-clusters might be podosomes, (2) that they may function as a basis for producing podosomes, and/or (3) that N-WASP, cortactin, Tks4, and Tks5 might be used in both actin-pl-clusters and podosomes. Indeed, in platelets, actin nodes/asters (called nodules) linking multiple actin filaments coexist with podosomes [62], although the nodules were much larger than the actin-pl-clusters. Therefore, although the original term “pl” in the phrase “actin-pl-clusters” stands for fluorescent phalloidin and Lifeact, the “pl” could now include the additional meaning of “podosome-like.”

The short residency times of N-WASP, Tks4, and Tks5 at the actin-pl-clusters (in the order of ~0.03‒0.3 s) indicate that the molecules in actin-pl-clusters exchange with those in the bulk cytoplasm rapidly, perhaps rendering the actin-pl-clusters responsive to the required changes of the actin nodes and podosome-like structures. Therefore, the next step of this research would be to elucidate how the actin-pl-clusters at the steady state are utilized for specific cellular responses upon stimulation, particularly for the initial engagement of podosome formation in migratory and invasive cells. If some of the actin-pl-clusters actually work in podosomes and invadosomes, then the actin-pl-clusters would play key roles in the mechanical transduction and cellular movements based on podosomes and invadosomes.

(5) The reason why large actin nodes/asters were not detected by 3D EM tomography is now clear. These structures do not exist in intact cells. However, why were the actin-pl-clusters not observed by electron tomography? This is probably because these structures are small, necessitating increased magnifications to visualize them, but this makes the observation view field quite small, and since the number of actin-pl-clusters is very limited, only 0 or 1 might be present in a view field, making the detection of actin-pl-clusters extremely difficult. Furthermore, since the actin-pl-cluster might be quite small, with a size less than 20 nm in diameter as described in the Introduction, differentiating actin-pl-clusters from many intersections of overlapping actin filaments in the layered actin meshwork would be very difficult.

We believe that the structures, dynamics, and molecular compositions of cortical actin filaments, including the fine actin meshwork and stress fibers, and particularly the actin-pl-clusters found in this work (true nodes for linking actin filaments in the cortical fine actin filament meshwork), should be considered in broad areas of studies of cellular functions, particularly in the research of cell motility, mechano-responses of cells, and cancer metastasis. Furthermore, as they are extremely concentrated on the PM cytoplasmic surface (within 3.5 nm from the surface), such structures must play important roles in the PM functions, including endocytosis, exocytosis, signal transduction, and cytokinesis.

## Supporting information

S1 Fig Actin-pl-clusters observed using SRM without arrowheads or over-contrasting. In Fig 2, all of the actin-pl-clusters found in the view field were indicated by magenta arrowheads, which are reproduced in this figure (left column). The same images without arrowheads are presented so that the images could be clearly inspected by readers. In Fig 2B, to make the fine actin meshwork visible, the contrast was over-enhanced (also shown here; middle column). Here, its under-contrasted images (no saturation in the images) are also shown (right column).
(TIF)

S2 Fig Actin-pl-clusters observed using SRM and TIRFM. In Fig 5, all of the actin-pl-clusters found in the view field were indicated by magenta arrowheads, which are reproduced in this figure (top row). The same images without arrowheads are shown here (bottom row) so that the images could be clearly inspected by readers.
(TIF)

S3 Fig Actin-pl-clusters visualized by GFP-UtrCH using TIRFM. **(Top)** A representative snapshot from image sequences of NRK cells transfected with GFP-UtrCH and observed by TIRFM. **(Middle row)** Snapshot images from two-color TIRFM observations of NRK cells cotransfected with GFP-UtrCH (green) and Lifeact-Halo labeled with TMR (red). **(Bottom row)** Snapshot images from two-color TIRFM observations of NRK cells cotransfected with GFP-UtrCH (green) and Lifeact-TM labeled with SeTau647 (red).
(TIF)

S4 Fig Western blot results, confirming Halo-filamin A expression. Control NRK cells (WT) and NRK cells transfected with Halo-filamin A (WT + Halo-filamin A) were subjected to western blot analyses. The expression of Halo-filamin A was difficult to detect using anti-filamin A polyclonal antibodies, probably because its expression level was much less than that of endogenous filamin A and also because the molecular weights of these two molecules are very close **(Top-left)**. However, the expression of Halo-filamin A was detected by using anti-Halo polyclonal antibodies **(Top-right)**. The results with an anti-α-tubulin monoclonal antibody **(Bottom-left)** and an anti-β-actin monoclonal antibody **(Bottom-right)** are shown as controls for the protein amounts.
(TIF)

S1 Movie Dynamic morphological changes of Actin-pl-clusters. Live-cell SRM observation of Lifeact-mGFP in an NRK cell, using the SDSRM of an Olympus SD-OSR system operated at a temporal resolution of 2 Hz (with a signal integration time of 0.5 s) for a period of 50 s. The scale bar indicates 5 μm.
(AVI)

S2 Movie Dynamic morphological changes of Actin-pl-clusters 2. Live-cell SRM observation of Lifeact-mGFP in an NRK cell, using the 3D-SIM mode of a Nikon N-SIM system operated at a temporal resolution of 0.44 Hz (with a signal integration time of 0.1 s) for a period of 60 s. The scale bar indicates 5 μm.
(AVI)

S3 Movie Single-molecule behavior of Lifeact-TM. A representative two-color TIRFM observation of Lifeact-mGFP (green) and Lifeact-TM-ACP-Setau647 (red) at 60 Hz (16.7-ms time resolution). The scale bar indicates 5 μm.
(AVI)

S4 Movie Single-molecule behavior of N-WASP. A representative two-color TIRFM observation of Lifeact-mGFP (green) and Halo-N-WASP labeled with TMR (red) at 60 Hz (16.7-ms time resolution). The scale bar indicates 5 μm.
(AVI)

S5 Movie Single-molecule behavior of Tks4. A representative two-color TIRFM observation of Lifeact-mGFP (green) and Halo-Tks4 labeled with TMR (red) at 60 Hz (16.7-ms time resolution). The scale bar indicates 5 μm.
(AVI)

S6 Movie Single-molecule behavior of Tks5. A representative two-color TIRFM observation of Lifeact-mGFP (green) and Halo-Tks5 labeled with TMR (red) at 60 Hz (16.7-ms time resolution). The scale bar indicates 5 μm.
(AVI)
