# Supplementary figures and images for: Cortical actin nodes: Their dynamics and recruitment of podosomal proteins as revealed by super-resolution and single-molecule microscopy
Source: PLoS One. 2017 Nov 30;12(11):e0188778. doi: 10.1371/journal.pone.0188778 (PMC5708734; doi:10.1371/journal.pone.0188778)

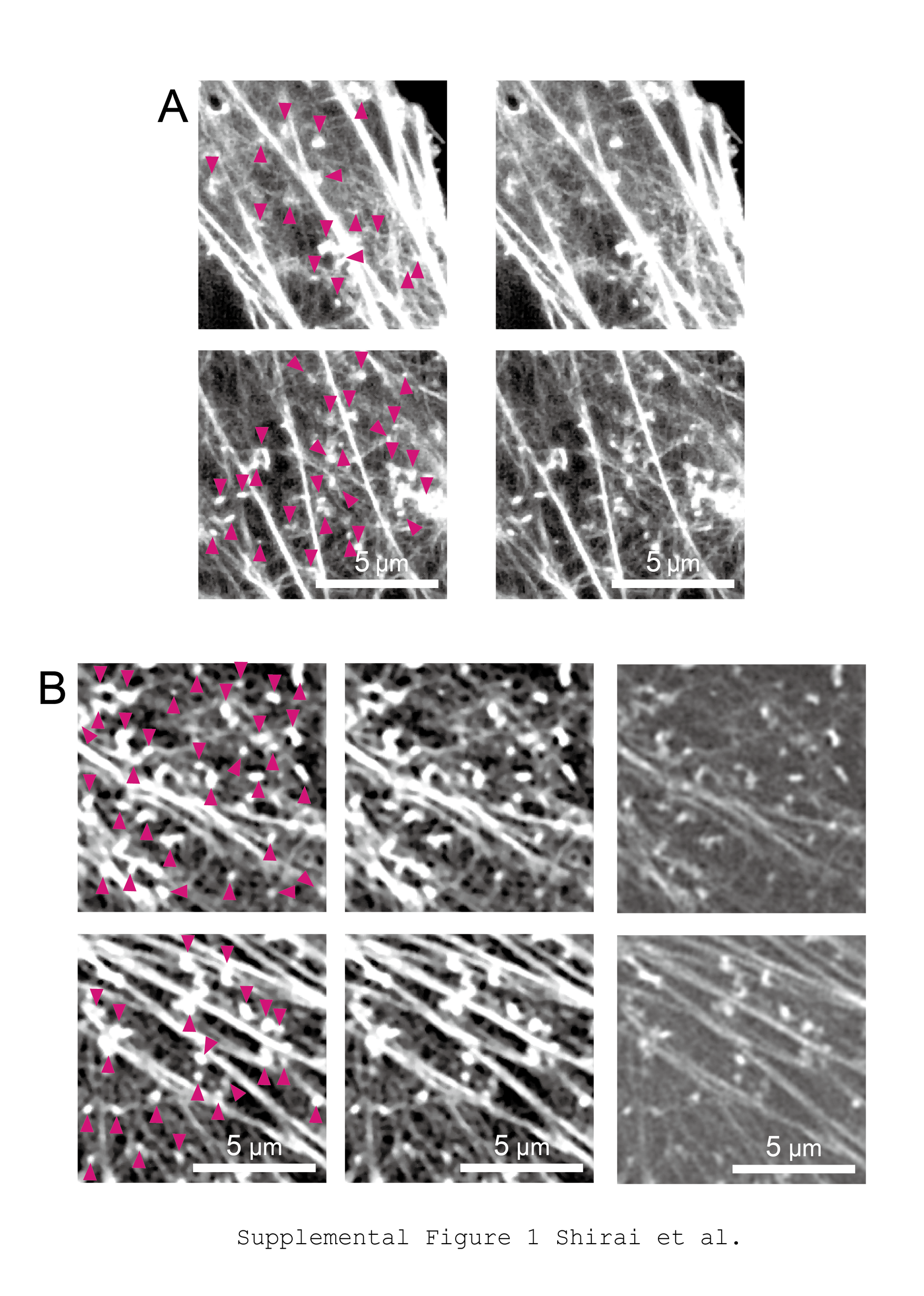

Supplement: S1 Fig — In Fig 2, all of the actin-pl-clusters found in the view field were indicated by magenta arrowheads, which are reproduced in this figure (left column). The same images without arrowheads are presented so that the images could be clearly inspected by readers. In Fig 2B, to make the fine actin meshwork visible, the contrast was over-enhanced (also shown here; middle column). Here, its under-contrasted images (no saturation in the images) are also shown (right column). (TIF) [file pone.0188778.s001.tif]

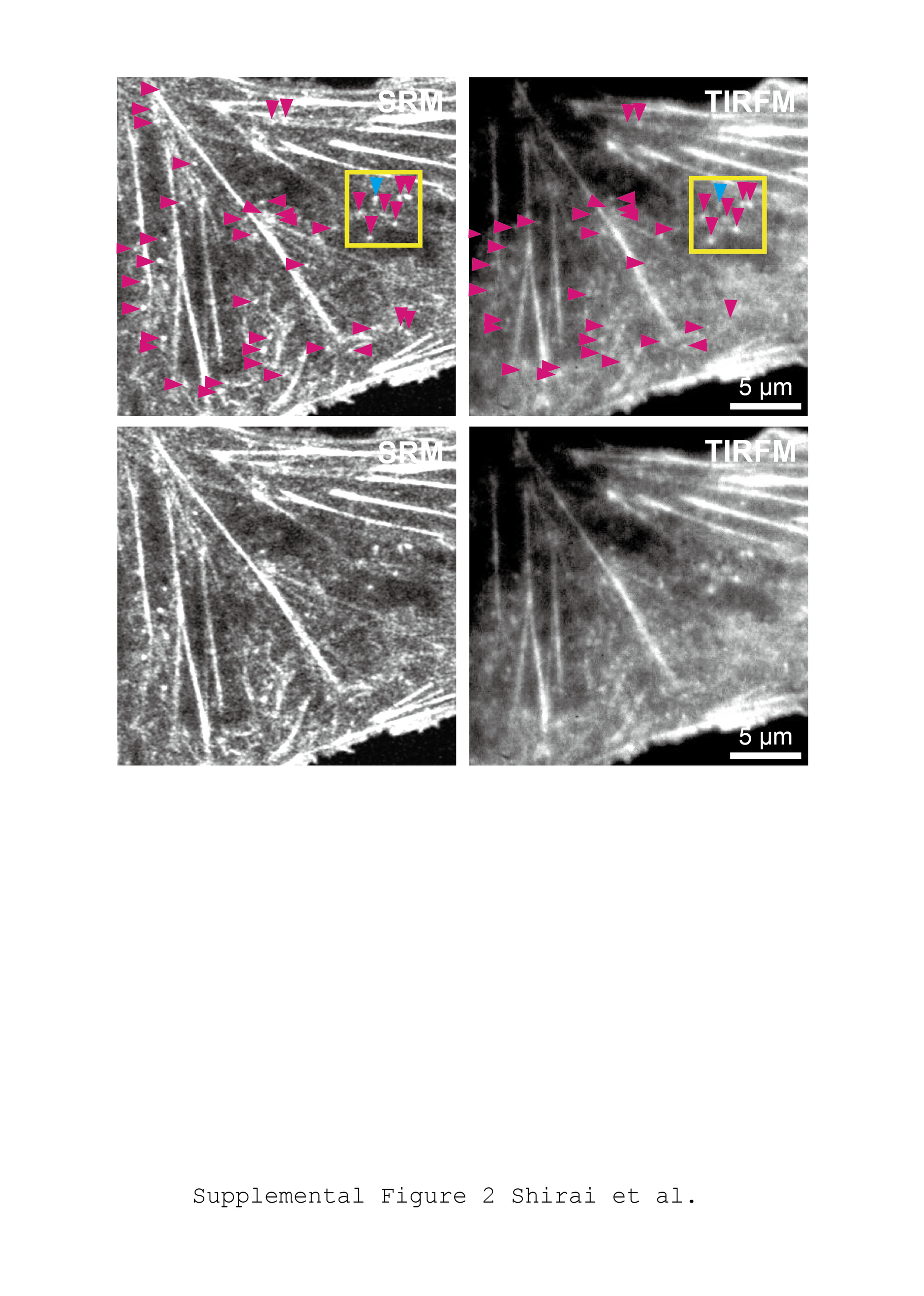

Supplement: S2 Fig — In Fig 5, all of the actin-pl-clusters found in the view field were indicated by magenta arrowheads, which are reproduced in this figure (top row). The same images without arrowheads are shown here (bottom row) so that the images could be clearly inspected by readers. (TIF) [file pone.0188778.s002.tif]

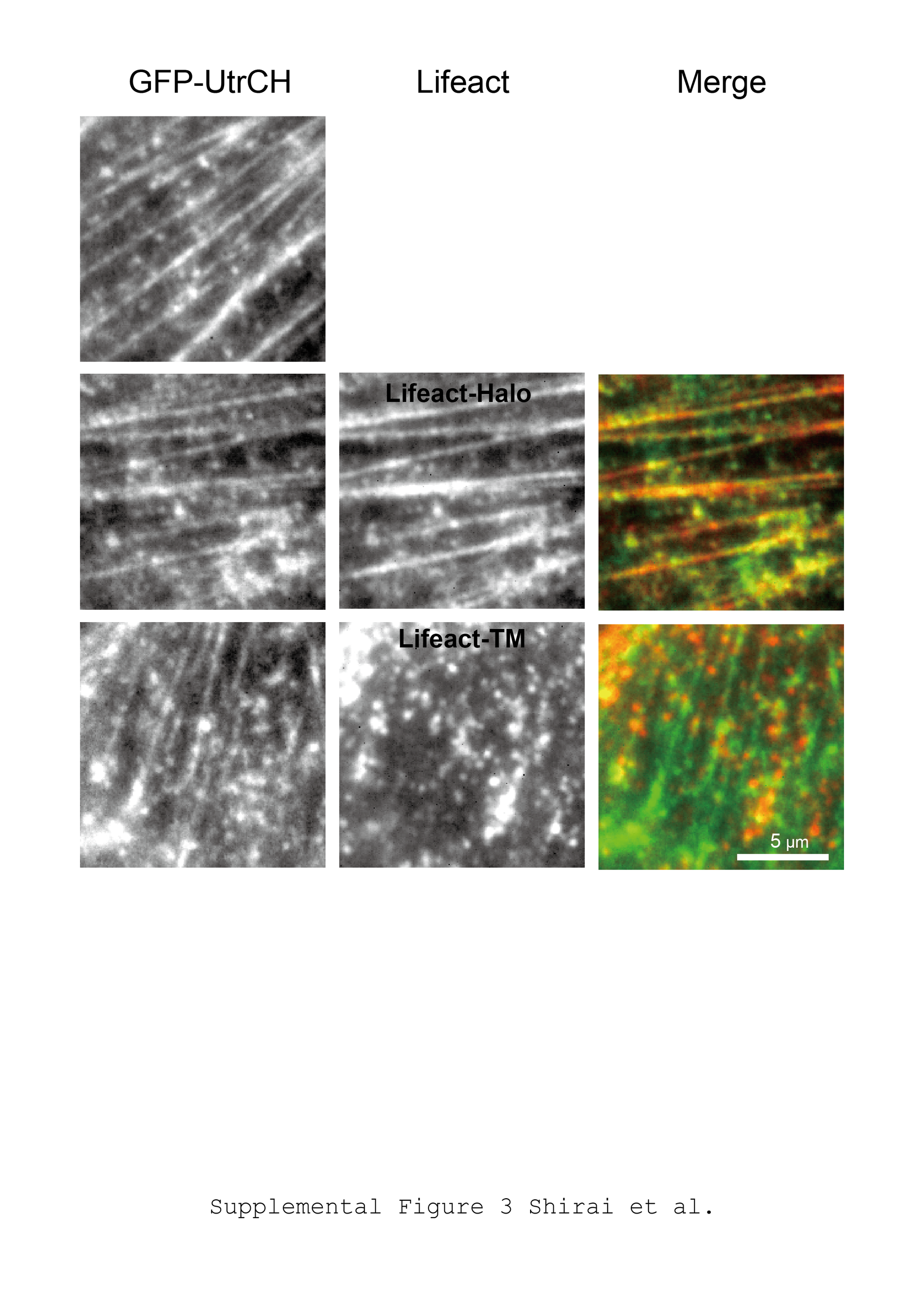

Supplement: S3 Fig — (Top) A representative snapshot from image sequences of NRK cells transfected with GFP-UtrCH and observed by TIRFM. (Middle row) Snapshot images from two-color TIRFM observations of NRK cells cotransfected with GFP-UtrCH (green) and Lifeact-Halo labeled with TMR (red). (Bottom row) Snapshot images from two-color TIRFM observations of NRK cells cotransfected with GFP-UtrCH (green) and Lifeact-TM labeled with SeTau647 (red). (TIF) [file pone.0188778.s003.tif]

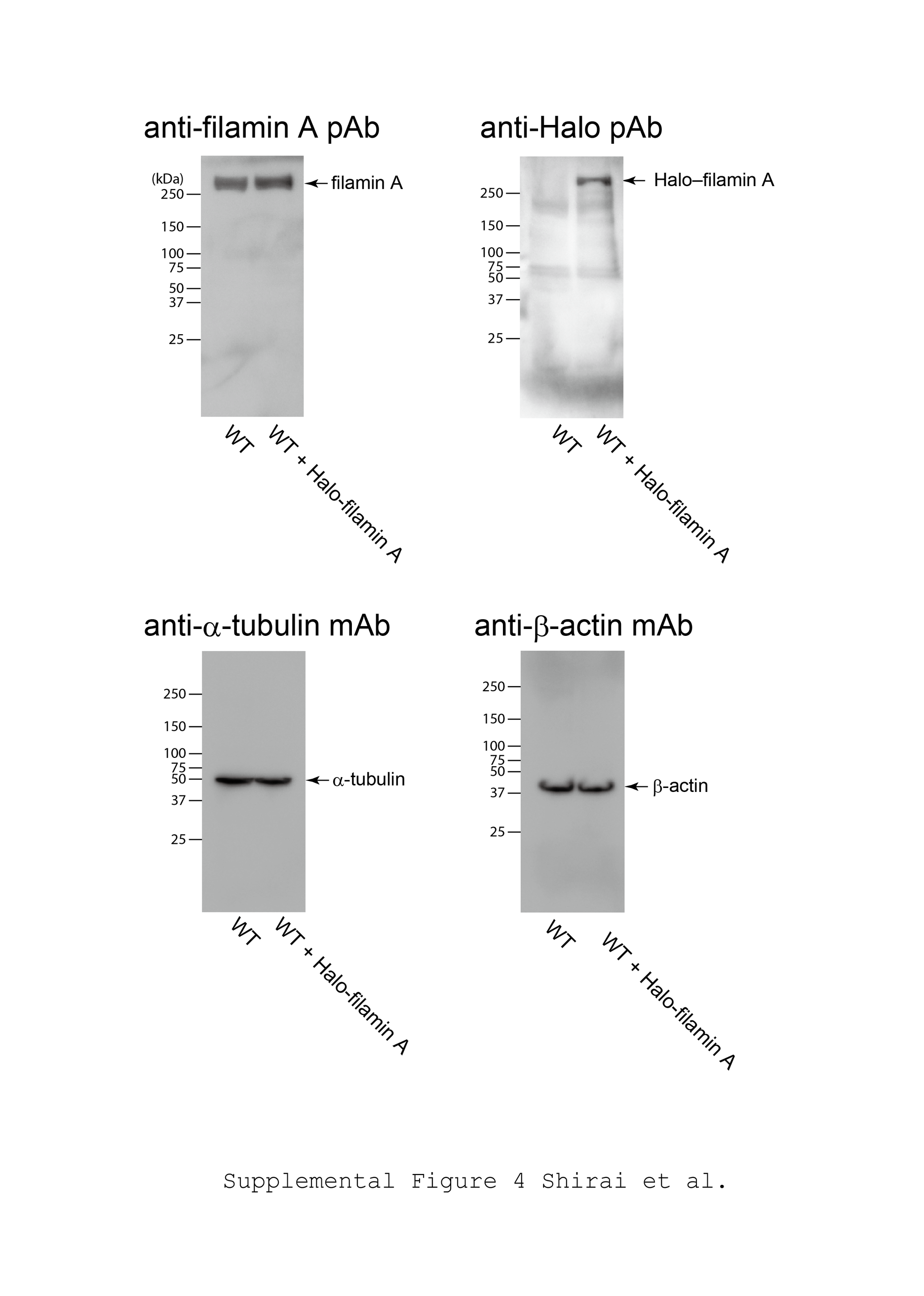

Supplement: S4 Fig — Control NRK cells (WT) and NRK cells transfected with Halo-filamin A (WT + Halo-filamin A) were subjected to western blot analyses. The expression of Halo-filamin A was difficult to detect using anti-filamin A polyclonal antibodies, probably because its expression level was much less than that of endogenous filamin A and also because the molecular weights of these two molecules are very close (Top-left). However, the expression of Halo-filamin A was detected by using anti-Halo polyclonal antibodies (Top-right). The results with an anti-α-tubulin monoclonal antibody (Bottom-left) and an anti-β-actin monoclonal antibody (Bottom-right) are shown as controls for the protein amounts. (TIF) [file pone.0188778.s004.tif]
